# A glycomics and proteomics study of aging and Parkinson’s disease in human brain

**DOI:** 10.1038/s41598-020-69480-3

**Published:** 2020-07-30

**Authors:** Rekha Raghunathan, John D. Hogan, Adam Labadorf, Richard H. Myers, Joseph Zaia

**Affiliations:** 1 Graduate Program in Molecular and Translational Medicine, Boston University School of Medicine, Boston, 02118 USA; 2 Department of Biochemistry, Boston University School of Medicine, 670 Albany St., Rm. 509, Boston, 02118 USA; 3 Bioinformatics Program, Boston University Graduate School of Arts and Sciences, Boston, 02118 USA; 4 Department of Neurology, Boston University School of Medicine, Boston, 02118 USA

**Keywords:** Glycomics, Proteomic analysis

## Abstract

Previous studies on Parkinson’s disease mechanisms have shown dysregulated extracellular transport of α-synuclein and growth factors in the extracellular space. In the human brain these consist of perineuronal nets, interstitial matrices, and basement membranes, each composed of a set of collagens, non-collagenous glycoproteins, proteoglycans, and hyaluronan. The manner by which amyloidogenic proteins spread extracellularly, become seeded, oligomerize, and are taken up by cells, depends on intricate interactions with extracellular matrix molecules. We sought to assess the alterations to structure of glycosaminoglycans and proteins that occur in PD brain relative to controls of similar age. We found that PD differs markedly from normal brain in upregulation of extracellular matrix structural components including collagens, proteoglycans and glycosaminoglycan binding molecules. We also observed that levels of hemoglobin chains, possibly related to defects in iron metabolism, were enriched in PD brains. These findings shed important new light on disease processes that occur in association with PD.

## Introduction

The volume of the extracellular space (~ 20%) that separates brain cell surfaces and through which molecules diffuse displays regional patterns that change during development, aging and neurodegeneration^1,2^. The passage of protein molecules through the extracellular space depends on the geometries and chemical compositions of extracellular and cell surface molecular complexes, the specific binding domains thereof, and the fixed negative charges of glycosaminoglycan chains^3,4^. Brain extracellular matrix (ECM) is composed of perineuronal nets (PNNs), interstitial matrices, and basement membranes (blood brain barrier), each consisting of a network of glycoproteins, proteoglycans, hyaluronan and collagens^5^. Despite the obvious importance of the extracellular space to neural plasticity and neurodegeneration^6,7^, there is little information available on the alterations that occur to these molecules during Parkinson’s disease (PD).

Inflammation and disruption of the blood brain barrier can lead to infiltration of fibroblasts and trigger a fibrotic response in an attempt to restore normal function^8^. Such fibrosis demolishes the structure of the ECM, and impedes healing by secreting inhibitory molecules and serves as a barrier to axons. Infiltration of fibroblasts leads to deposition of thrombin and fibrinogen and destruction of the integrity of the ECM. These inflammatory reactions lead to local neural degeneration and activation of glial cells. In PD, the activation of glial cells and recruitment of T-cells leads to increased pro-inflammatory cytokine release and increased levels of reactive oxygen and nitrogen species. While disruption is not believed to occur, activated microglia appear to induce blood brain barrier dysfunction in PD^9^. Despite this, limited information is available concerning the changes in the distribution of ECM molecules in PD, with the exception of glycosaminoglycans (GAGs) found in senile plaques and Lewy bodies^10,11^.

α-Synuclein comprises 1% of cytosolic protein in the nervous system, participates in synaptic transmission, and is modulated in conditions that alter neural plasticity^12^. Its pathology is prevalent in different PD brain regions. The Braak six-stage scheme, based on α-synuclein immunohistochemistry in autopsy cases, follows an ascending course that starts with the olfactory bulb and culminates in widespread pathology in cortical regions^13,14^. In PD brain, the majority of abnormal α-synuclein deposits occur in neuritic processes. This aberrant deposition drives PD pathogenesis.

The molecular actors in secretion and internalization of α-synuclein remain undefined. Endocytosis of α-synuclein from the extracellular space by astrocytes results in its degradation, a process that becomes overloaded in PD, resulting in accumulation of α-synuclein in the cytosol^14^. The presence of GAGs in Lewy bodies in PD patients suggests a role in the accumulation of α-synuclein. In particular, GAGs may bind proteases and impede degradation of α-synuclein^15^. Heparan sulfate proteoglycans (HSPGs) appear to participate in uptake and seeding of α-synuclein through micropinocytosis^16,17^. There may also be a connection to impairment of lysosomal degradation of GAGs^18^.

Glycosaminoglycans have been found in all types of extracellular amyloid deposits^19,20^. Heparan sulfate (HS) proteoglycans sequester pro-inflammatory molecules, regulate recruitment of leucocytes to the inflammation/injury site and participate in internalization of amyloid aggregates^16,21–23^. Amyloid aggregates in Alzheimer’s disease (AD) and PD brain are thought to spread through prion-like seeding mechanisms involving binding interactions with GAGs and other ECM components^17^. As part of this mechanism, cell surface HSPGs and chondroitin sulfate proteoglycans (CSPGs) are involved in uptake of α-synuclein aggregates. Recent results have shown that internalization of such aggregates in neuroblastoma cells is HS-dependent but that of smaller non-amyloid oligomers is not^12^.

We observed in a rat brain aging study that overall abundance of HS in striatum diminished with age and the relative abundance of sulfated domains increased^24^. In substantia nigra, by contrast, the overall HS abundance was unchanged and the relative abundances of sulfated domains decreased. Thus, the HS chain structure was brain-region specific, as were age-related changes to HS structure. These observations led us to examine the correlations of GAG and protein abundances in human brains with PD versus those with no neurological disease. Since the nigrostriatal pathway becomes impacted heavily by time of death, we chose to analyze prefrontal cortex tissue specimens. We now present the results of glycomics and proteomics analysis of two separate human brain tissue cohorts. Our results identify changes to the HS and CS domain structures correlated with aging and PD. The most highly enriched protein gene set in PD was ECM proteins, consisting of collagens, hyalectan proteoglycans, HAPLNs and fibrinogens. Notably, a set of four hemoglobins, including α, β, γ1 and γ2 were also enriched in PD brains. We discuss the results and implications for understanding of PD mechanisms. We compare our human brain aging results against our previously published rat brain aging study^24^.

## Methods

De-identified frozen brain tissue specimens from Brodmann area 9 were acquired from the National Brain Tissue Resource for Parkinson’s Disease and Related Disorders at Banner Sun Health Research Institute, Sun City, AZ^25^ and the Harvard Brain and Tissue Resource Center, McLean Hospital, Belmont, MA. The ranges in age, post mortem intervals (PMIs), and RNA integrity numbers (RINs) are shown in Table 1. The PMI range for cohort 1 was lower than for cohort 2. The RIN numbers were similar for the two cohorts, indicating that all biospecimens were of high quality. The available clinical data on the biospecimens are shown in Supplemental Table 1. The experimental protocols were performed in accordance with US National Institutes of Health guidelines on human subjects research. The protocol for use of banked de-identified human brain tissue was reviewed by the Boston University Medical Campus Institutional Review Board and determined not to meet the definition of human subjects research.

**Table 1:** Human brain cohorts.

	Samples	N	Age range (mean)	PMI range (mean)	RIN range (mean)
Cohort 2	Young	12	36–60 (49.8)	15–18 (22)	7.3–8.5 (7.8)
	Aged	13	64–97 (74.3)	2–32 (18)	6.4–9.1 (8.0)
	PD	16	65–74 (77.4)	7–31 (18)	6.1–8.0 (7.0)
Cohort 1	Aged	12	69–91 (81.2)	2–5 (2.4)	6.0–8.7 (8.0)
	PD	12	64–88 (77.8)	2–4 (2.5)	6.1–8.5 (7.2)

### Brain tissue cryosectioning

Fresh frozen human brain tissue blocks were mounted on chucks using optimal cutting temperature (OCT) polymer. Coronal tissue sections (10 µm thick) were cut from the tissue not in contact with OCT using a cryostat at – 20 °C. Sections were adhered to Superfrost Plus microscope slides and submerged in acetone for one min. at − 20 °C. We used our published method to apply enzymes to the surfaces of tissue slices mounted on slides^26^. Briefly, one 5 mm diameter grey matter area on the slide surface was selected per brain as shown in the photographs in Figure Supplementary Fig. S1A. Some of the photographic images are of low resolution. We marked the digestion spots on the back of the slide using solvent-resistant ink. We used the ink spot to guide application of enzyme droplets.

### Sample preparation for glycomics and proteomics

Two cohorts of male human brain prefrontal cortex biospecimens were analyzed (Table 1). Clinical information on the biospecimens is shown in Supplementary Table S1. We selected grey matter areas of Brodmann area 9 with the knowledge that PD-related processes would be evident given the radiating nature of the spread of the disease in brain. Grey matter contains numerous cell bodies and few myelinated axons. White matter is composed primarily of myelinated axon tracts. We used our technique whereby glycans and proteins are released by serial digestion with small droplets of enzyme solution, corresponding to a spot of about 5 mm, from the surface of a tissue slide^24,27,28^. This allowed us to select grey matter areas accurately. The average distance between a neuron and a microvessel in grey matter is ~ 20 µm^29^, meaning that our technique sampled numerous vessels, extracellular space, and cellular interfaces. We quantified the abundances of proteins from proteomics data set and used GO annotations to assign them to compartment.

Fresh frozen human brain frontal cortex Brodmann area 9 slides were prepared for three serial sections per brain specimen. The slides were processed using our published method^26^ as summarized in Supplementary Fig. S1A. Briefly, all samples were blinded and processed in random order. Tissue slides were immersed in a series of ethanol washes (100%, 90%, 70%, 50%, 30%, water) and then digested using a series of glycosidase enzymes followed by trypsin as described^24,26^. The slides were digested first using five cycles of hyaluronidase and the digestion products were extracted using a dilute ammonium hydroxide solution. The process was repeated using chondroitinase ABC digestion, and then heparin lyase I, II and III digestion. The tissue slides were then reduced, alkylated, digested using trypsin, and the tryptic peptides extracted. Extracted glycan solutions were desalted using size exclusion chromatography. The grey matter regions selected for each specimen are shown in Supplementary Fig. S1B.

### Glycomics

Saccharides released using chondroitinase and heparinases, respectively, were analyzed using in-house packed nano-flow HILIC columns interfaced with an Orbitrap XL mass spectrometer operating in negative polarity mode as described^24,26,30^. Isomeric disaccharides were differentiated using on-line tandem MS. The disaccharide LC–MS peak areas were normalized relative to an internal standard and the relative peak areas were calculated. Box whisker plots were generated using the Realstats add-in for the Microsoft Excel spreadsheet software^31^.

### Proteomics

Label free proteomics data were acquired as described previously^24,26^. Briefly, a Q-Exactive HF mass spectrometer interfaced with a 150 µm C18 column was used with a 120 min gradient. Data were searched against the Uniprot/Swissprot database using Peaks Studio version 8.5 (Bioinformatics Solutions, Inc., Waterloo, ON, Canada). The following variable modifications were used: deamidation N, oxidation M, phosphorylation STY, HexNAc ST, HexHexNAc ST, hydroxylation K, hydroxylation-Hex K, ubiquitination K, hydroxylation P, nitrotyrosine Y. A maximum of 2 missed cleavages, a 10 ppm precursor ion and 0.1 Da product ion mass tolerance were specified. Principal component analysis (PCA) was carried out using the PEAKs proteins.csv export files using our in-house PEAKSviz software^32^. For Cohort 1, two groups were specified: aged (> 60 years, no neurological disease) and PD (> 60 years). For Cohort 2, three groups were specified: young (< 60 years, no neurological disease), aged (> 60 years, no neurological disease) and PD (> 60 years. The lists of differentially abundant proteins were analyzed using gene set enrichment analysis (GSEA)^33^ using the WebGestalt gene set analysis toolkit^34^. For aged versus PD comparisons, the list of differentially abundant proteins present in both cohorts was used for GSEA.

## Results and discussion

### Brain biospecimen integrity

The concern for human brain biospecimen studies is that PMI, disease states and effects of medication may bias the conclusions reached. Studies of gene expression in post-mortem human brain have established the critical importance of minimal RNA degradation in biospecimen quality. Despite its widespread use by brain tissue resource centers to assess biospecimen quality^35^, it must be acknowledged that RIN values reflect incomplete measures of brain biospecimen quality^36–38^. While proteins are considered to be reasonably stable in brain post-mortem^39,40^, it remains possible that molecular changes to proteins could conceivably bias proteomics results. While the average PMIs for cohort 1 were higher than those for cohort 2, biospecimen RIN numbers for the two cohorts were similar (Table 1). We present our results with the acknowledgement that the only way to eliminate all possible sources of bias related to tissue procurement and processing is to repeat the work using separate biospecimen cohorts in future studies.

### Glycomics

**Figure 1:**
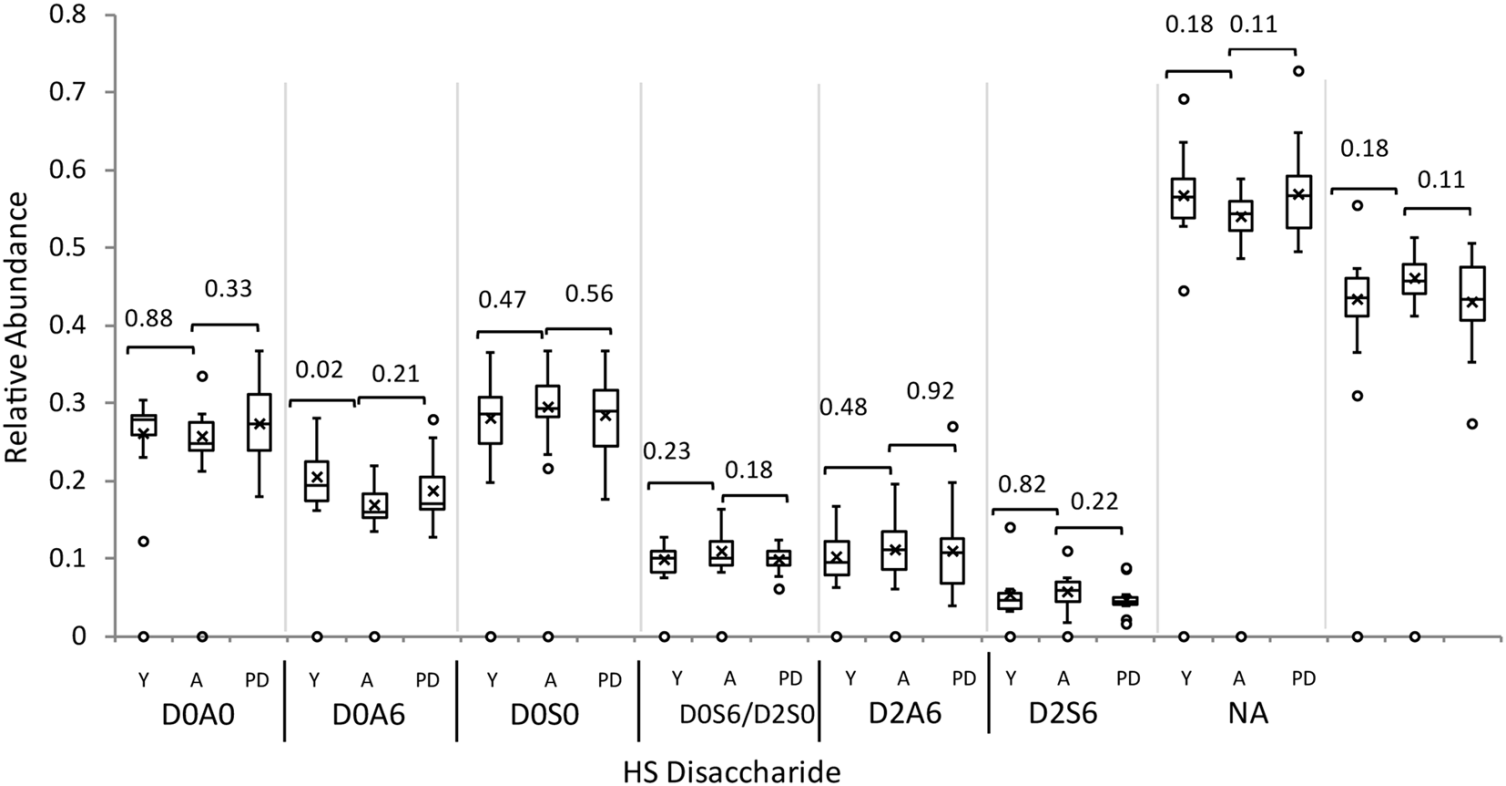
HS glycomics of human brain prefrontal cortex for young (Y), aged (A) and Parkinson’s disease (PD) brain from cohort 2. Box plots with outliers showing disaccharide abundances, omitting D2A0 which was below the limits of detection. Uncorrected p values from two tailed T tests are shown. D0A0 ΔHexAGlcNAc, D0A6 ΔHexAGlcNAc6S, D0S0 ΔHexAGlcNS, D0S6 ΔHexAGlcNS6S, D2A6 ΔHexA2SGlcNAc6S, D2S6 ΔHexA2SGlcNS6S, *NA* sum of *N*-acetylated disaccharides, *NS* sum of *N*-sulfated disaccharides. Detailed structures are shown in Supplementary Fig. S3. Whiskers show maximum and minimum values. The top and bottom of each box show the 75th and 25th percentile of the sample, respectively. The line through each box shows the median and the x marker the mean of the samples.

**Figure 2:**
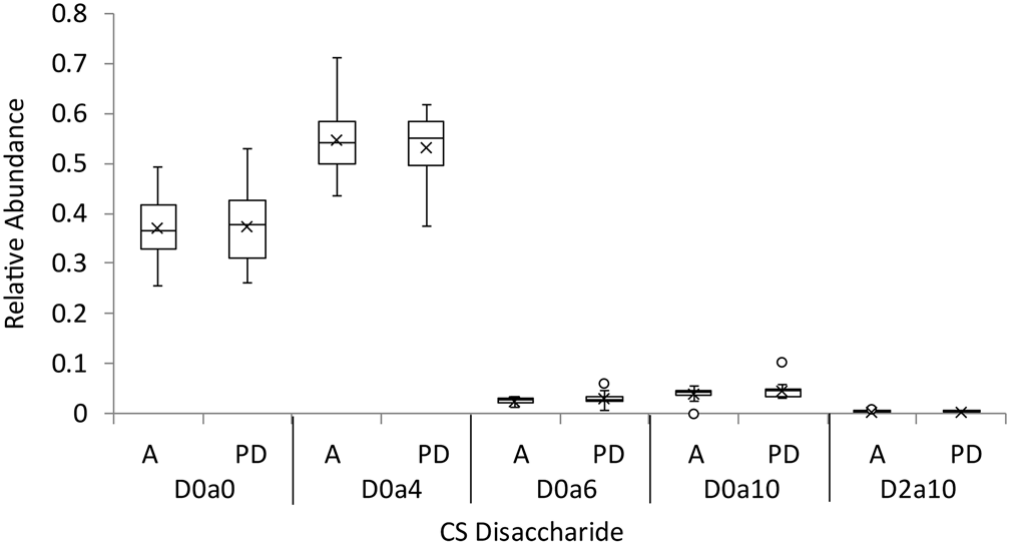
CS glycomics of human brain prefrontal cortex aged with no neurological disease (**A**) and aged with Parkinson’s disease (PD) from cohort 1. CS Disaccharide abundances, D0a0 ΔHexAGalNAc, D0a4 ΔHexAGalNAc4S, D0a6 ΔHexAGalNAc6S, D0a10 ΔHexAGalNAc4S,6S. Detailed structures are shown in Supplementary Fig. S4. Whiskers show maximum and minimum values. The top and bottom of each box show the 75th and 25th percentile of the sample, respectively. The line through each box shows the median and the x marker the mean of the samples.

Heparan sulfate chains on cell surface and extracellular proteoglycans bind numerous families of growth factors, morphogens and receptors^41^. Polymerized as nascent chains by a series of biosynthetic enzymes in the Golgi apparatus, mature HS chains consist of domains of highly sulfated disaccharide units (S domains), lowly sulfated disaccharide units (A domains) mixed (S/A) domains^42^ (Supplementary Fig. S2). Heparin lyase enzymes cleave the HS chains into disaccharides, see the structures and abbreviations for which in Supplementary Fig. S3. We measured the abundances of HS disaccharides released from cohort 2 tissue slide surfaces using heparin lyase enzymes. Cohort two contained three biological groups, young (< 60 years) with no neurological disease, aged (> 60 years) with no neurological disease, and aged with PD. Figure 1 shows box whisker plots of the abundances of HS disaccharides released by heparin lyase enzymes from brain cortex slides from cohort 2, for young (< 60 years), aged (> 60 years), and PD (> 60 years) individuals. As is typical for brain cortex^24,43^, the HS chains showed abundant unsulfated D0A0 and *N*-sulfated D0S0 disaccharides. The order of abundances for *N*-acetylated disaccharides was D0A0 > D0A6 > D2A6 > D2A0, and that for *N*-sulfated disaccharides D0S0 > D0S6/D2S0 > D2S6. The combined abundances of *N-*acetylated (NA) and *N*-sulfated (NS) disaccharides are also shown. The uncorrected individual two tailed t-test p values are given in the figure. These results are consistent with alterations in HS chain architecture during adult aging in brain cortex. Different HS alterations appear to occur in PD versus unaffected cortex. Specifically, the abundances of D0A6 diminished with aging. By contrast, the abundances of NA domains increased and NS domains decreased, with PD relative to controls. We analyzed CS chains from cohort 1 brain cortex specimens (Fig. 2). The structures of CS disaccharides released by chondroitinase ABC digestion are shown in Supplementary Fig. S4. Cohort 1 consisted of two groups, aged with no neurological disease and aged with PD. As shown in Fig. 2, the CS disaccharide abundances were similar between the two groups.

### Proteomics

We used LC–MS label-free proteomics to quantify tryptic peptides released from the surfaces of brain tissue microscope slides. This technique typically quantifies a few hundred proteins and is useful for comparing enrichment of protein gene sets among a sample cohort. By comparison, the MALDI imaging technique^44^ produces higher spatial resolution but does not produce direct protein identification and is less useful for protein gene set analysis.

#### Proteomics changes with age

To place our proteomics results for human brain in context, we show GSEA of data from our rat aging proteomics study^24^ for which strong positive enrichment with age in striatum of the organic acid binding gene set (GO: 0043177, Supplementary Table S2A, Supplementary Fig. S5) that included HAPLN1, versican and brevican. In light of these observations, it is interesting that that PNN form during the early post-natal period in striatum in mice^45^. By contrast, in substantia nigra, the most confident result was strong negative enrichment of the ribosomal structural constituents gene set (GO: 0003735, Supplementary Table S2B, Supplementary Fig. S6). These results demonstrated that brain region-specific patterns of enriched protein gene sets during aging.

Immunohistochemical mapping in rat brain has shown much higher levels of PNN in striatum than in substantia nigra^46^. In our previously published study, HAPLN1, versican (CSPG2), brevican (PCGB) and tenascin-R (TENR) were each more abundant in aged rat striatum, Supplementary Fig. S7. By contrast, in rat substantia nigra, versican, brevican and tenascin-R abundances each did not change; however, HAPLN1 increased in abundance with age. That PNN-associated molecules are present in both striatum and substantia nigra is consistent with the conclusion that the differences in PNN immunohistochemical staining levels^46^ reflected the abundance of particular molecular epitopes, rather than the level of the entire ECM macromolecule.

**Figure 3:**
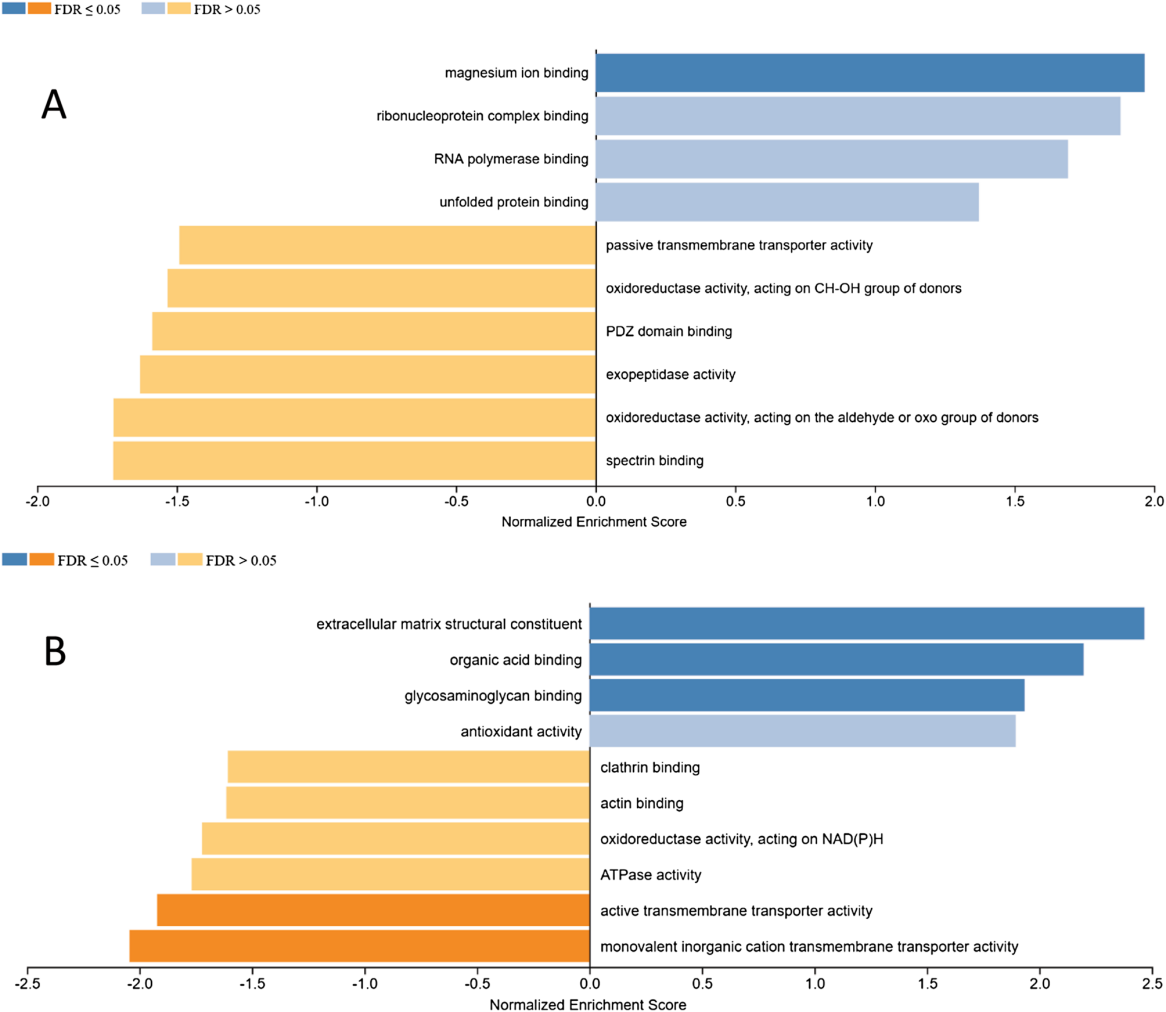
(**A**) GSEA of differentially expressed proteins from cohort 2 young (< 60 years) versus aged (> 60 years). (**B**) GSEA of differentially expressed proteins from cohorts 1 and 2 aged (> 60 years) versus PD (> 60 years).

In our human studies, we used grey matter areas from prefrontal cortex Brodman areas 9. Cohort 2 included unaffected individuals ranging in age from 36 to 97 years. No juveniles were included in this cohort and we make no attempt to compare adult aging with juvenile brain. Grouping the unaffected individuals based on the age of incidence of sporadic PD (young < 60 years, aged > 60 years), GSEA of the proteomics data indicated that the most confident result was enrichment of proteins in the magnesium binding gene set (Fig. 3A, GO: 0000287, Supplementary Table S3, Supplementary Fig. S8). In this gene set, enolase 1 and enolase 2 were strongly enriched with age. Known as neuron-specific enolase, enolase 2 is expressed at very high levels in neurons and neural tissue and is cited as a biomarker for neurological injury^47–49^. Enolase 1 is known as non-neuronal enolase because it is found in several tissues, including brain. Enolases have been cited to move to the cell surface under inflammatory conditions and play roles in extracellular matrix related to neurodegeneration^50^, consistent with our results.

RAN is a small GTP binding protein member of the RAS family that participates in translocation of RNA and proteins through the nuclear pore complex. Disruption of subcellular protein localization in neurodegenerative diseases, possibly in response to oxidative stress, resulting from alterations in nuclear transport, are associated with RAN function^51^.

α-Synuclein, the function of which relates to control of vesicular neurotransmission^52^, was also enriched in aged human prefrontal cortex (Supplementary Table S3). This result is in contrast to the decrease in α-synuclein mRNA observed in aging mouse substantia nigra^53^; however, levels of oligomerized α-synuclein measured by ELISA increased with age in monkey brain^54^.

#### Proteomics changes with Parkinson’s disease

Proteomics data were acquired for control and PD specimens and separately for cohorts 1 and 2. The differential protein abundance t-values calculated using Peaks Viz were reasonably well-correlated, see Supplementary Fig. S9A. For proteins observed in both cohorts, the most confidently enriched gene set for the comparison of aged without neurological disease versus PD was extracellular matrix structural component, see Fig. 3B, (GO: 0005201, Supplementary Table S4A). This gene set was dominated by collagen types I, II, III and IV chains and PNN-associated proteoglycans and glycoproteins. From the published literature, gene expression for collagen type IV, a key component of basement membranes, was increased in transgenic mice overexpressing α-synuclein, consistent with a significant role for this collagen in α-synuclein toxicity^55^. Otherwise, the literature for the roles of collagens in the mechanism of PD is remarkably sparse. Our results are consistent with significant changes to fibrillar (types I, II and III) and basement membrane collagens (type IV) associated with PD. This set also included tenascin-C, a hexameric glycoprotein that links ECM molecules including phosphacan, neurocan, aggrecan, cytokines and morphogens^56^. The gene set also included fibrinogen chains. It is known that cultured astrocytes and neurons express fibrinogen chains, indicating that they can be produced endogenously in the brain^57^. We observed fibrinogen α, β, and γ in the enriched extracellular matrix structural component gene set, indicating that the chains were higher in abundance in PD for both cohorts.

Proteins from the organic acid binding gene set enriched in our unaffected aged versus PD human proteomics data, included hemoglobins α, β, γ1 and γ2 (Supplementary Table S4B). Hemoglobin chains expressed in dopaminergic neurons function in mitochondrial homeostasis and their potential roles in neurodegeneration related to iron metabolism have been identified^58,59^. Published evidence supports a correlation between the levels of brain hemoglobin and neurodegenerative diseases including PD^60–62^. It is important to emphasize that our cohorts consisted only of males and that brain mitochondrial hemoglobin levels differ significantly between males and females^63^. The presence of fetal hemoglobin γ chains in human brain tissue was reported in separate a proteomics studies^64,65^. Our observation that hemoglobin γ1 and γ2 chains were present in aged brain and enriched in PD is novel to the best of our knowledge.

The annexins are a family of proteins capable of binding anionic phospholipids in a calcium-dependent manner^66^. Glycosaminoglycan binding has been reported for annexins 4, 5 and 6^67^, each of which is included in the glycosaminoglycan binding gene set (GO: 0005539, Supplementary Table S5) that was enriched in our proteomics data (Supplementary Fig. S9D). The complete set of proteins identified in our data included annexins 1, 2, 4, 5, 6, 7, 11 (Supplementary Fig. S10). Of these annexins 4, 5, 6 and 11 had t values indicating increased abundances in PD. The similar enrichment of annexins 4, 5, 6 and annexin 11 leads us to speculate that annexin 11 also binds GAGs.

Two gene sets associated with transmembrane transport (GO: 0015077, 58 proteins, Supplementary Table S6 and G0: 0022804, Supplementary Table S7) were enriched in aged prefrontal cortex unaffected by neurological disease (Fig. 3B). These results are consistent with decreased levels of a large number of proteins that participate in transmembrane transport in PD brain. The most highly enriched member is synaptosome protein 25, the loss of which in cultured neurons is associated with Golgi abnormalities and cell death^68^. This gene set also included several members of the solute carrier family of membrane transport proteins, cytochrome oxidases, and ATP synthetases, consistent with the conclusion that the energetics of transmembrane transport in synapses are altered in prefrontal cortex of PD brains.

## Conclusions

The challenges with omics analysis of human brain tissue are several-fold. Because these were human samples, there was high inherent genetic, proteomic and glycomic variability. Therefore, in order to identify the molecular alterations specific to the disease, we used the largest economically-feasible number of specimens to achieve statistical significance^69^. We chose to process the frozen brain biospecimens as tissue slides. One advantage to this approach, whereby enzymes are applied to the tissue slide surface and the released molecules analyzed using LC–MS, is that the effort required for sample cleanup prior to LC–MS analysis is modest. Another advantage is that we were able to analyze consistent tissue morphologies among all samples by applying the enzymes to equivalent grey matter areas visible on the slides. By comparison, MALDI methods for tissue imaging provide higher spatial resolution but do not identify proteins directly^44^. MALDI methods for profiling enzymatically released glycans from microscope slides achieve high spatial resolution^70^ but are not appropriate for quantification of HS and CS saccharides because these compounds dissociate during the MALDI process. Therefore, our LC–MS profiling approach provides a unique combination of appropriate throughput and analytical sensitivity to quantify HS, CS and proteins among a comparatively large set of brain specimens.

Our workflow is unique in combining both glycomics and proteomics profiling from brain tissue. HS and CS polysaccharides are expressed with pattern of highly sulfated domains interspersed with domains of low sulfation^42^. The compositions and structures of such domains vary according to cell type, location, development, and disease state, indicative of the growth factor binding characteristics of extracellular microenvironment^71^. Therefore, alterations in GAG disaccharide profiles with disease indicate changes in the manner in which signaling proteins are presented to cellular receptors. We show the first disaccharide analysis results from human brain tissue for PD versus control specimens. These results indicate changes in the biosynthetic processes and/or expression of proteoglycan core proteins associated with PD. Our results also show that changes in HS disaccharide composition that occur with human aging are distinct from those that occur in PD. Because we used only one cohort for the HS analyses, it will be necessary to acquire data on additional and larger specimen cohorts in order to rule out all potential sources of bias.

Our proteomics results are significant because they were collected on a defined cortex region (Brodmann area 9) and morphology (grey matter). The most appropriate way to gauge the biological significance of these proteomics results is by grouping proteins into gene sets using GSEA. This technique identifies the gene sets that are most enriched in the proteomics data to maximize the likelihood of statistical significance. The time efficiency of our on-slide digestion and analysis method^26^ allowed us to acquire proteomics data on two separate sample cohorts of 24 and 41 specimens, respectively, a relatively large number considering the challenges of working with wet tissue.

Our goal was to identify the brain tissue alterations that occur in glycosaminoglycans and proteins associated with normal aging in contrast with those that occur in PD. For human aging proteomics, the magnesium ion-binding molecular functions gene set was the most confidently enriched. The most highly enriched set members included RAN, enolase 1 and enolase 2. α-Synuclein was also a member of this gene set. By contrast, the previously published rat brain aging proteomics study^24^ showed enrichment of ribosome structural components for substantia nigra and organic acid binding genes for striatum. It should be noted that the rat study compares juvenile versus aged individuals and the results are best judged in that context. The human study cohort 2 compares ages ranging from 36–97 years but does not include juveniles. Therefore, the human brain aging study highlights changes to the prefrontal cortex grey matter proteome that occur with aging in the absence of neurological disease.

For the comparison of PD versus normal aging, extracellular matrix proteins were the most confidently enriched, including collagens, HAPLNs, hyalectans, tenascins and fibrinogens. ECM changes are among the upregulated gene sets enriched in an mRNASeq analysis and are enriched in Huntington’s disease^72^. Our findings are consistent with these studies. We conclude from our proteomics results from two specimen cohorts that changes in extracellular matrix occur in PD brain. We also observed four hemoglobin chains were enriched in PD prefrontal cortex, all of which have been identified in published human brain proteomics datasets. The unexpected observation that fetal hemoglobin chains are enriched in PD is likely to be of mechanistic interest for future studies.

## Supplementary information

Supplementary information

## Data Availability

All data described here have been deposited in a public repository and will be made available upon publication. Proteomics data are available through the Pride Repository (dataset identifier PXD018736) Glycomics data are available through the GlycoPOST repository (dataset identifier GPST000033).
