# Supplementary material for: A glycomics and proteomics study of aging and Parkinson’s disease in human brain
Source: Sci Rep. 2020 Jul 30;10:12804. doi: 10.1038/s41598-020-69480-3 (PMC7393382; doi:10.1038/s41598-020-69480-3)
Supplement: Supplementary file 1 — Supplementary information [file 41598_2020_69480_MOESM1_ESM.pdf]

A glycomics and proteomics study of aging and Parkinson's disease in human brain

Rekha Raghunathan[1], John D. Hogan[3], Adam Labadorf[3], Richard H. Myers[1,3,4],

and Joseph Zaia[1,2,3,*]

[1] Boston University, Molecular and Translational Medicine, Boston, 02118, USA

[2] Boston University, Biochemistry, Boston, 02118, USA

[3] Boston University, Bioinformatics Program, Boston, 02118, USA

[4] Boston University, Department of Neurology, Boston, 02118, USA

Supplementary Material

Supplementary Figures……… p. 2

Supplementary Tables………..p. 20

Supplementary References…..p. 30

Supplementary Figure S 1. (A) Tissue omics workflow [1]. Fresh frozen brain tissue was cryosectioned and mounted on microscope slides. A 5 mm diameter droplet of enzyme solution was applied to the tissue surface. The slides were transferred to a humidified chamber and allowed to digest. The digestion products were extracted by pipetting. The digestion products were cleaned and then analyzed using LC-MS. The process was repeated for three glycosidases (hyaluronidase, chondroitinases, heparin lyases) and trypsin. Abbreviations: HA, hyaluronan; CS, chondroitin sulfate; HS, heparan sulfate; LC-MS, liquid chromatography-mass spectrometry; HILIC, hydrophilic interaction chromatography.

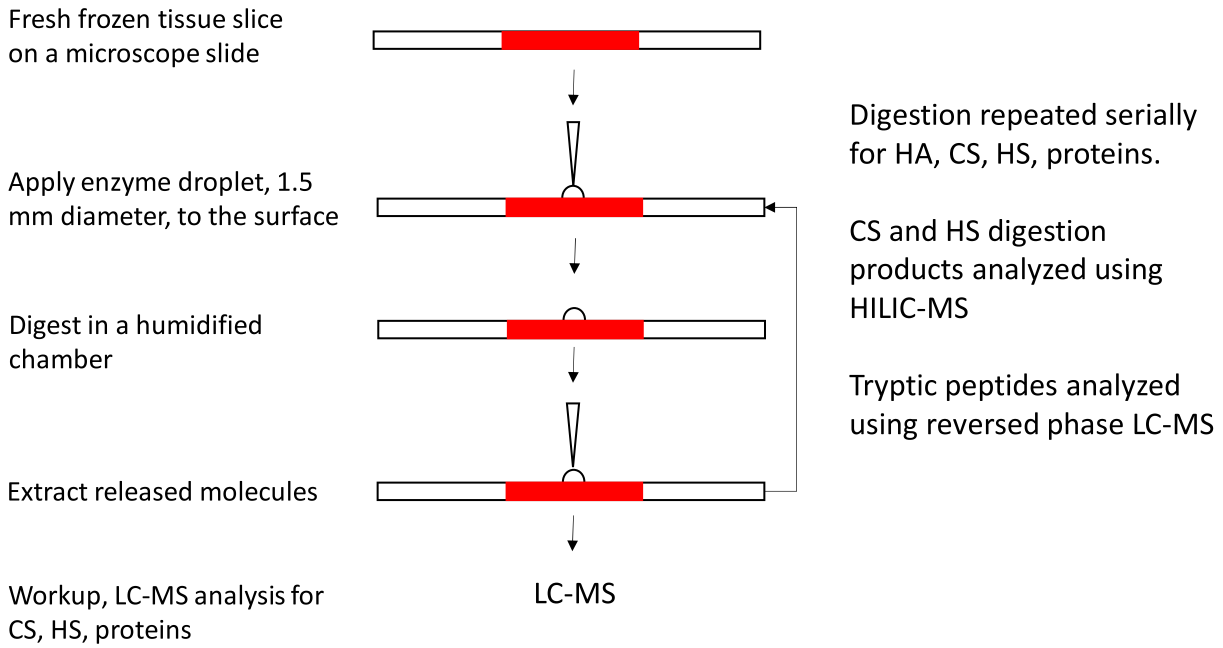

Supplementary Figure S 1B. Photographic images of the brain tissue blocks used in the aging and PD study, mounted on cryotome chucks. The 5 mm diameter grey matter areas to which enzyme digestion solution was applied on tissue slices prepared from these tissue blocks are marked with blue circles.

Cohort 1

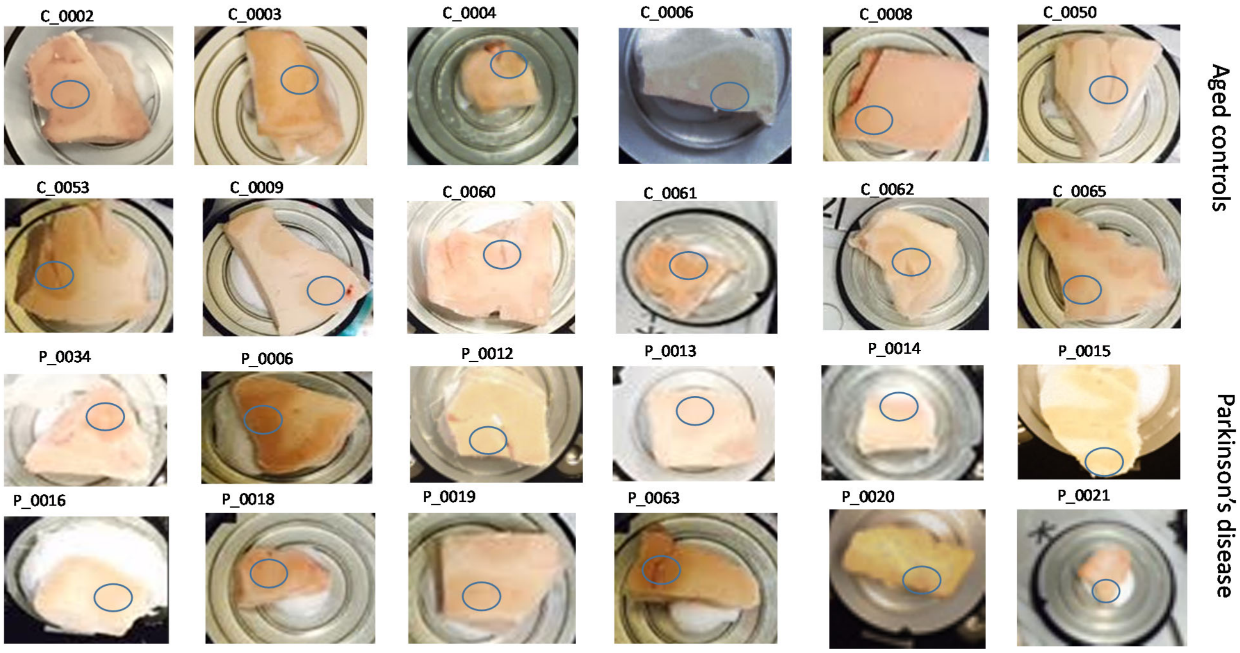

Supplemental Figure 1B (continued) Cohort 2

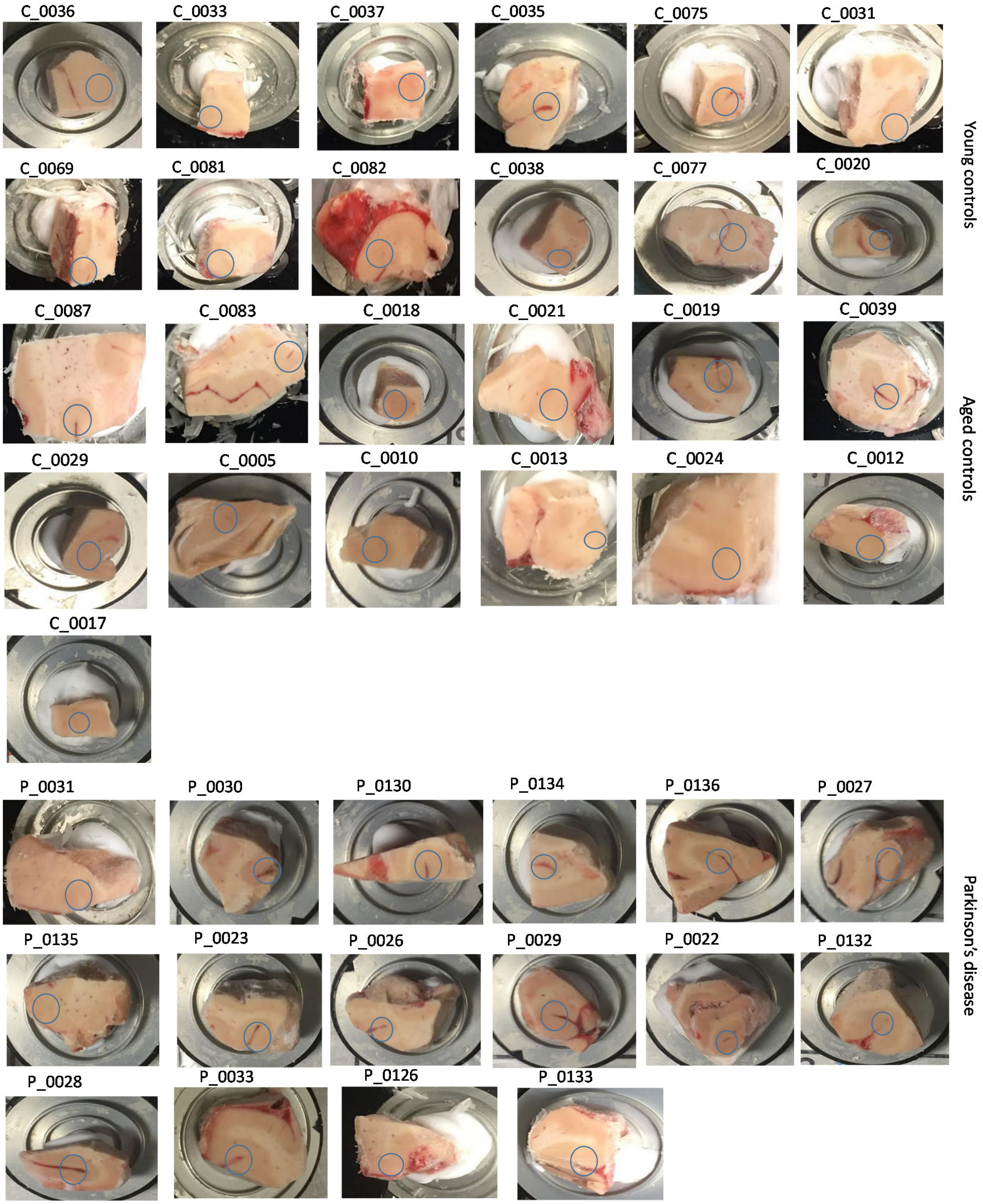

Supplementary Figure S 2. Biosynthesis of heparan sulfate (HS)[2]. Chains are biosynthesized by a series of enzymes in the endoplasmic reticulum and Golgi apparatus. The mature chains have a structure consisting of domains of high, low, and intermediate degree of sulfation. The structures and patterns of these domains varies in a spatially and temporally regulated manner[3].

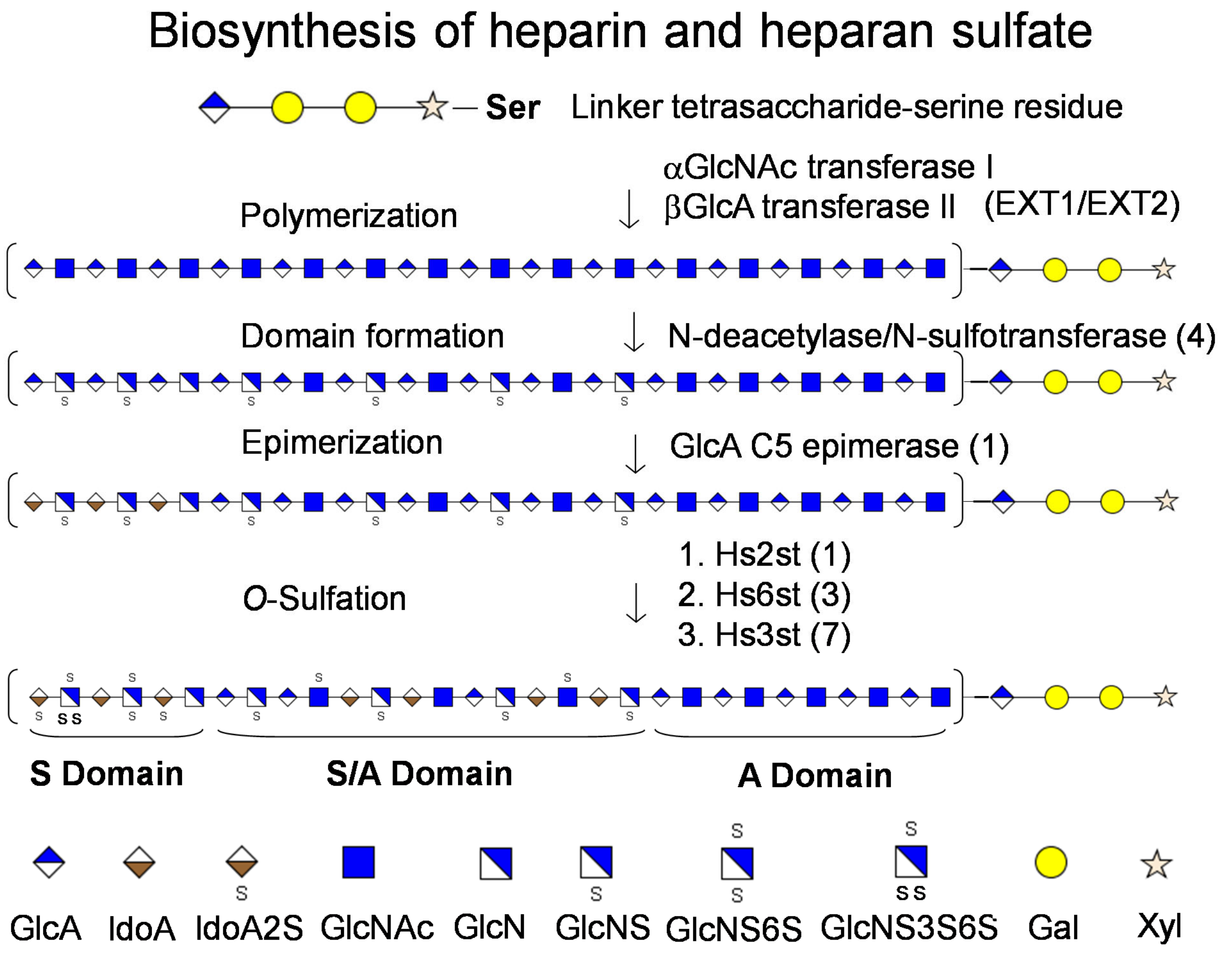

Supplementary Figure S 3. Nomenclature of HS disaccharides

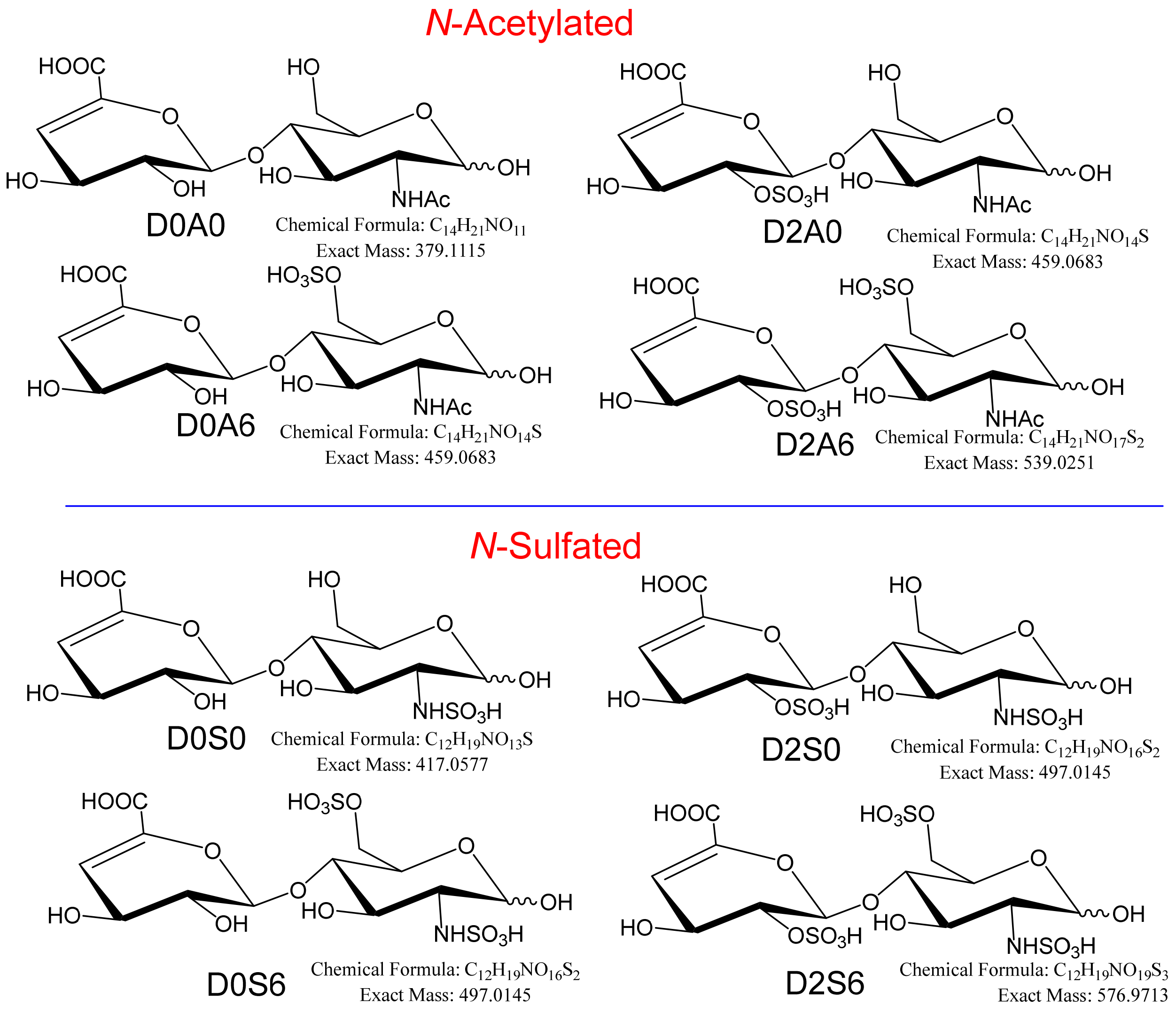

Supplementary Figure S 4. Nomenclature of CS disaccharides

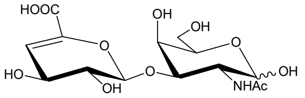

D0a0

$C_{14}H_{21}NO_{11}$
Exact Mass: 379.11

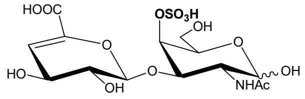

D0a4

$C_{14}H_{21}NO_{14}S$
Exact Mass: 459.07

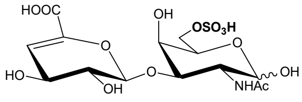

D0a6

$C_{14}H_{21}NO_{14}S$
Exact Mass: 459.07

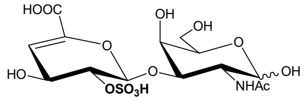

D2a0

$C_{14}H_{21}NO_{14}S$
Exact Mass: 459.07

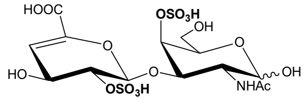

D2a4

$C_{14}H_{21}NO_{17}S_2$
Exact Mass: 539.03

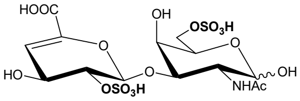

D2a6

$C_{14}H_{21}NO_{17}S_2$
Exact Mass: 539.03

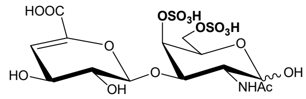

D0a10

$C_{14}H_{21}NO_{17}S_2$
Exact Mass: 539.03

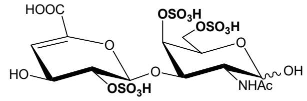

D2a10

$C_{14}H_{21}NO_{20}S_3$
Exact Mass: 618.98

Supplementary Figure S 5. GSEA of differentially expressed proteins young versus aged rat striatum from previously published data[4]. (A) Bar plot showing enriched gene sets.

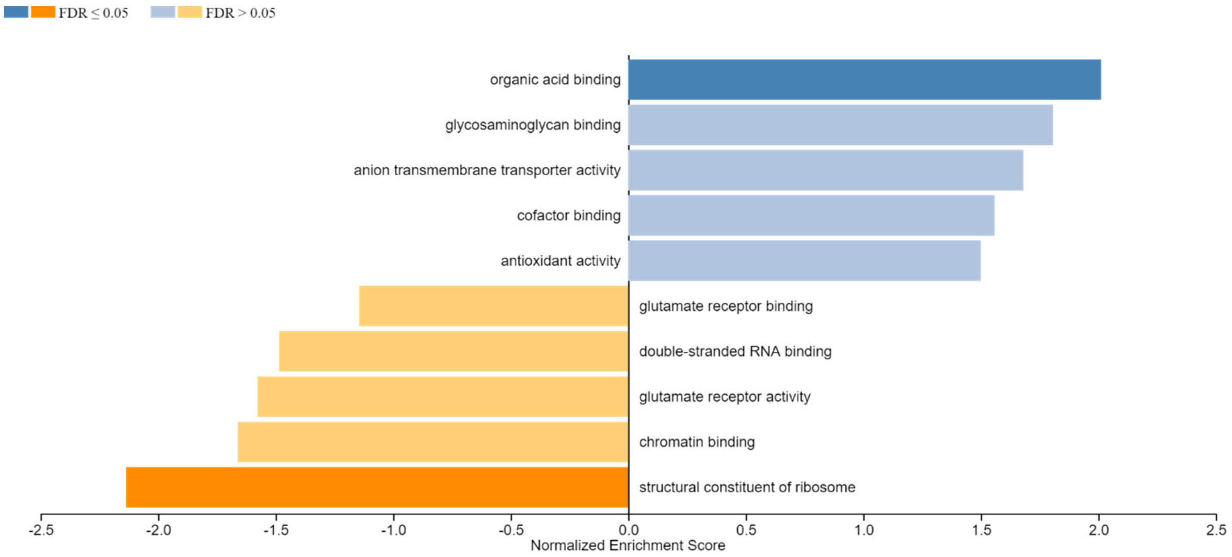

Supplementary Figure S 5. GSEA of differentially expressed proteins young versus aged rat striatum from previously published data[4]. (B) Volcano plot showing most enriched molecular functions.

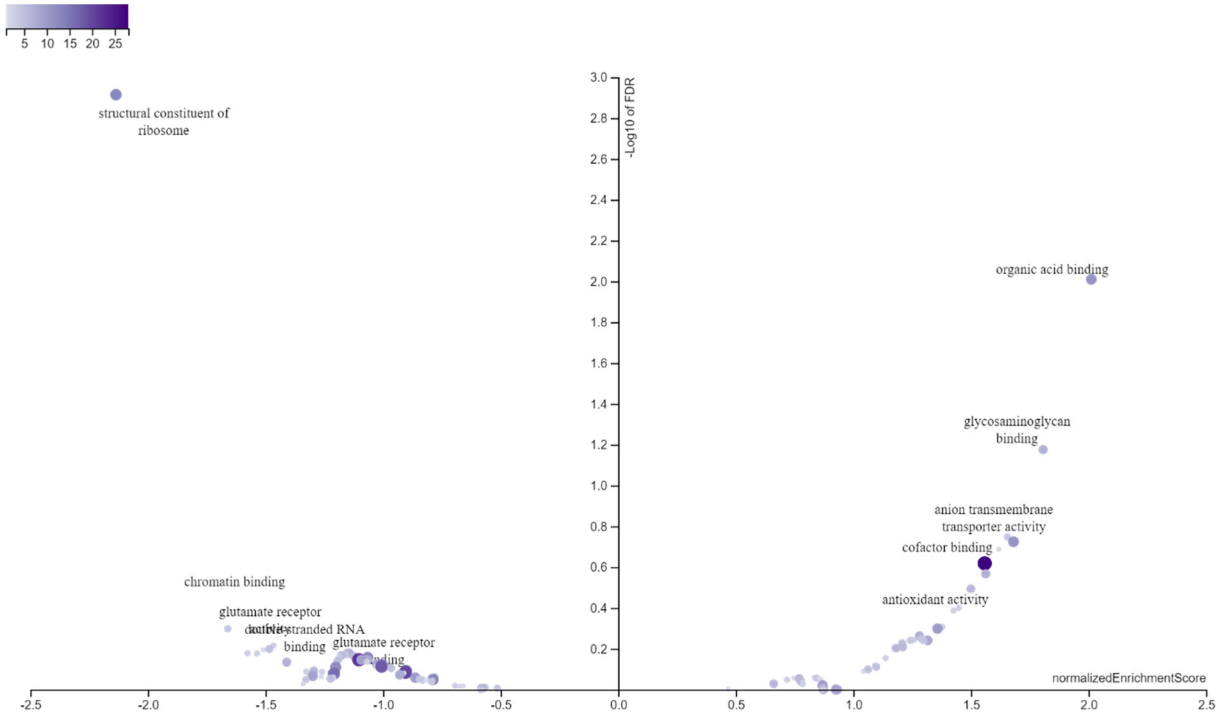

Supplementary Figure S 6. GSEA of differentially expressed proteins in young versus aged rat substantia nigra from previously published data[4]. (A) Bar plot showing enriched gene sets.

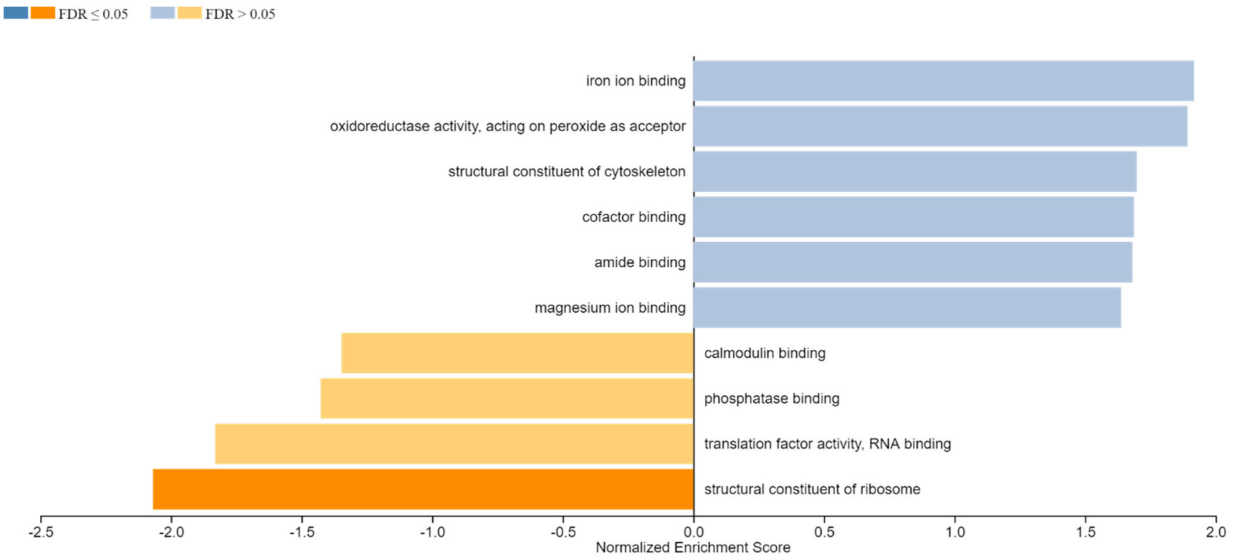

Supplementary Figure S 6. GSEA of differentially expressed proteins in young versus aged rat substantia nigra from previously published data[4]. (B) Volcano plot showing most enriched molecular functions.

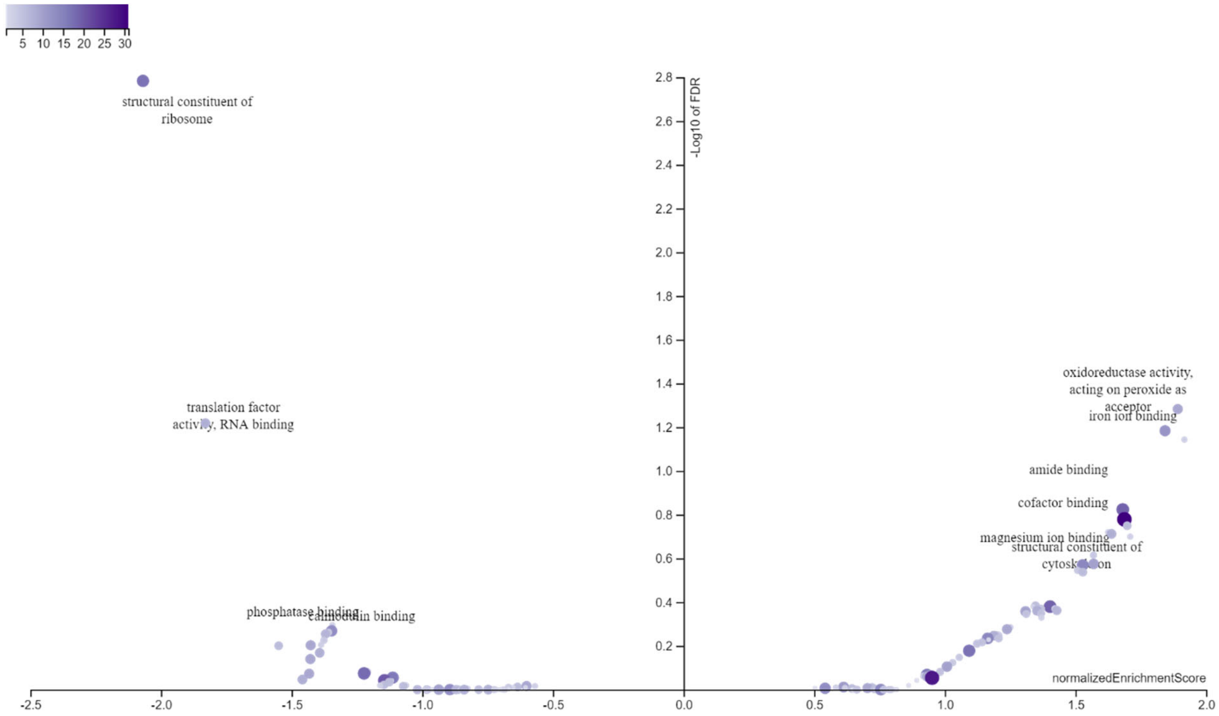

Supplementary Figure S 7**.** Comparison of PNN-associated protein abundances in young vs aged rat brain (A) striatum (STR) and (B) substantia nigra (SN) from previously published data[4].

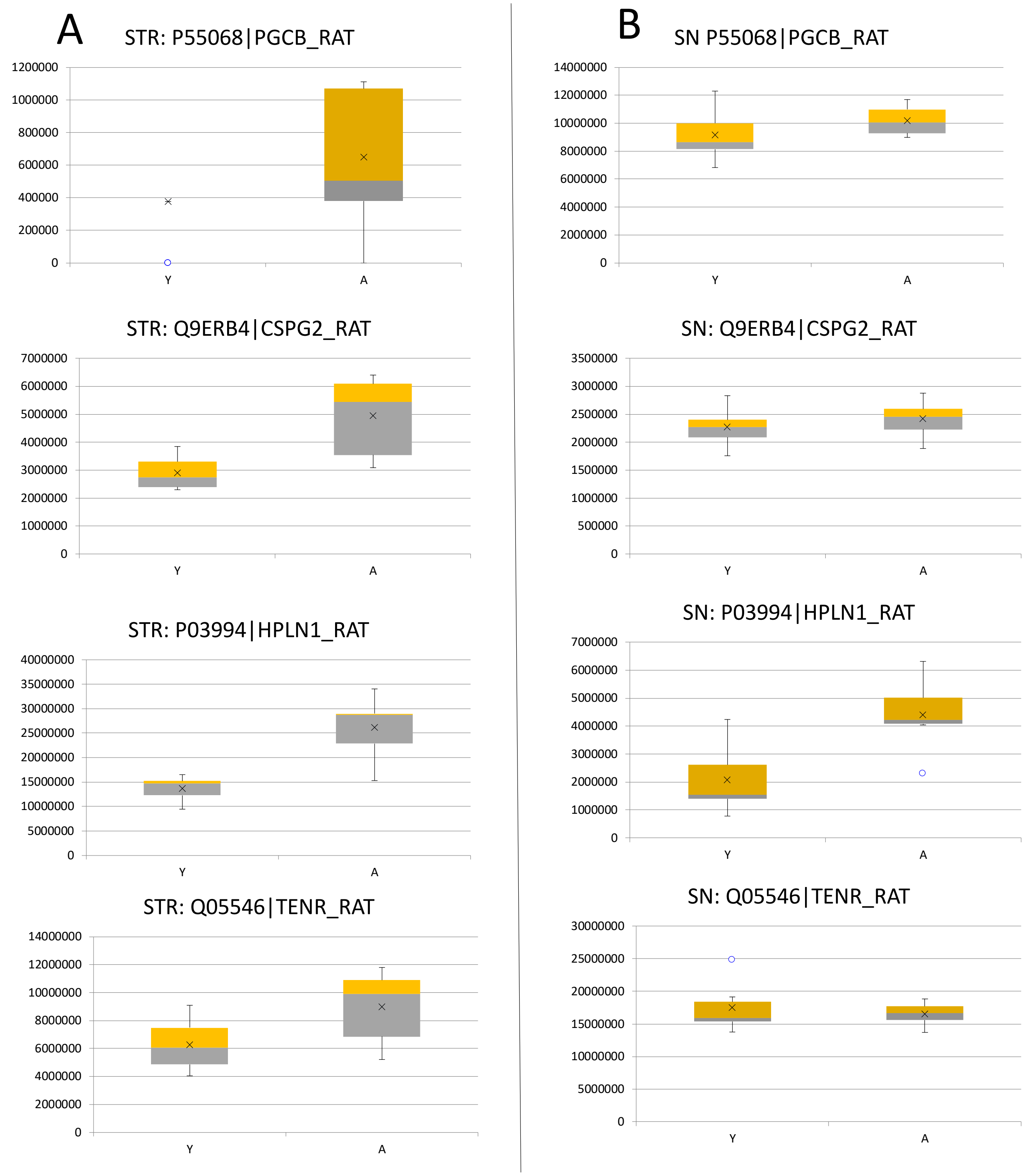

Supplementary Figure S 8. GSEA of differentially expressed human proteins young (<60 years) versus aged (>60 years). (A) Volcano plot showing most enriched molecular functions

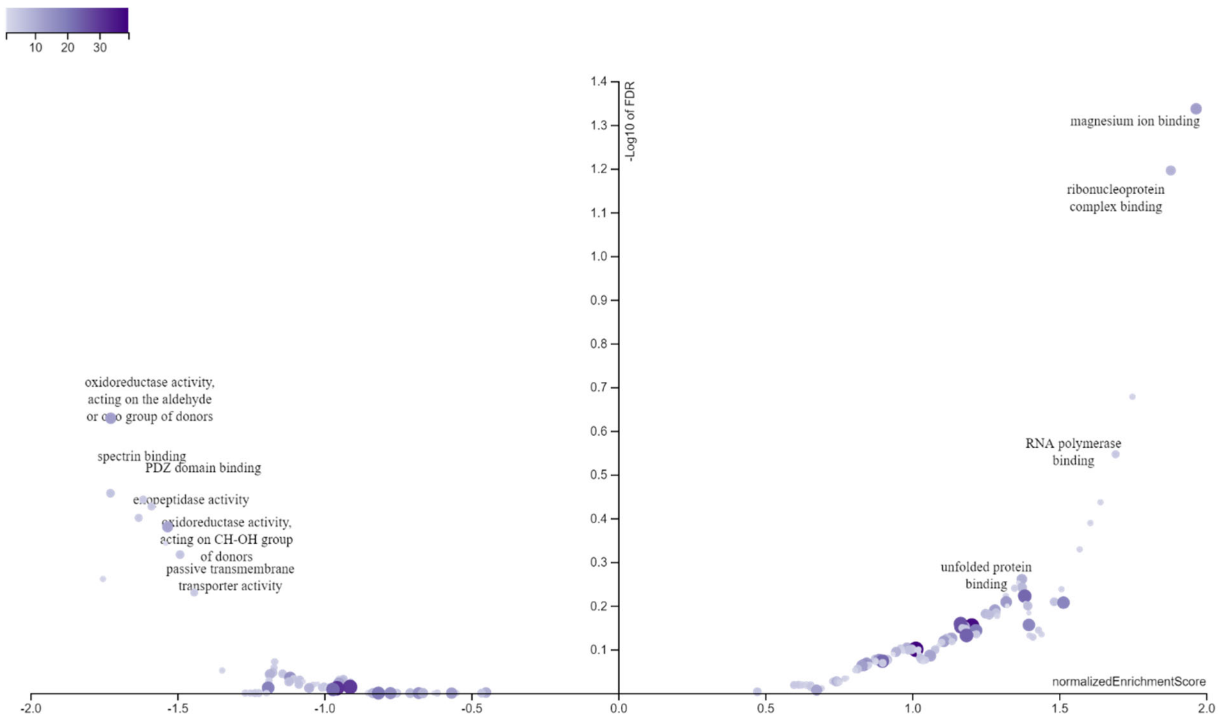

Supplementary Figure S 8. GSEA of differentially expressed human proteins young (<60 years) versus aged (>60 years). (B) Directed acyclic graph of GO terms.

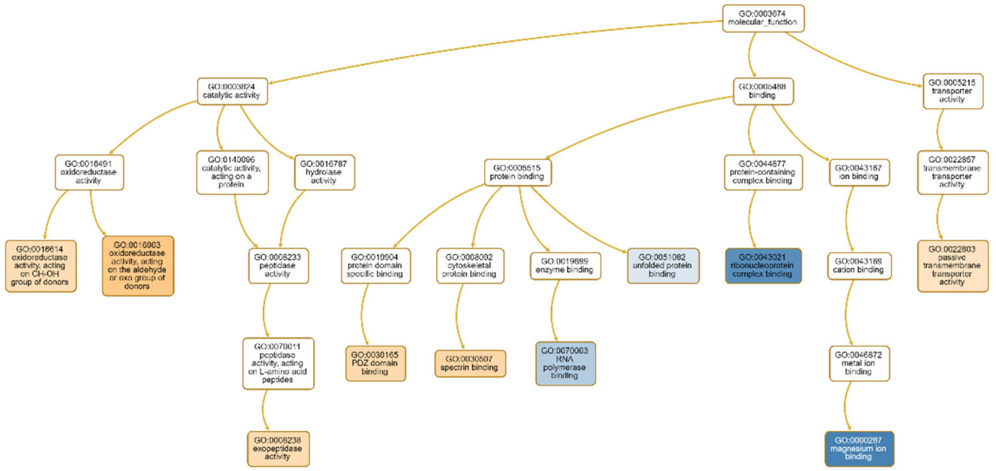

Supplementary Figure S 9**.** GSEA of differentially expressed human prefrontal cortex grey matter proteins observed in both cohort 1 and cohort, Aged (>60 years) versus PD,

(A) plot of differential abundance t-values for the two cohorts.

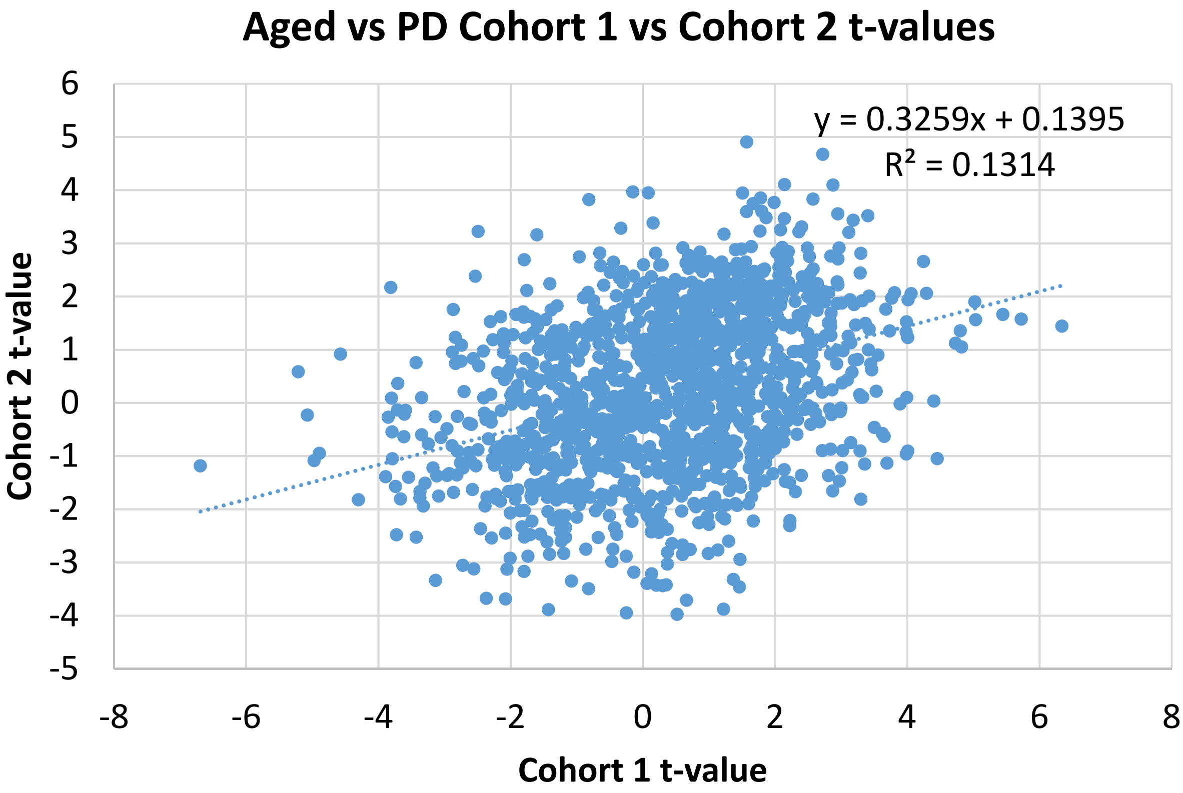

Supplementary Figure S 9. GSEA of differentially expressed human prefrontal cortex grey matter proteins observed in both cohort 1 and cohort 2, Aged (>60 years) versus PD,

(B) principal component analysis of cohort 1 and cohort 2

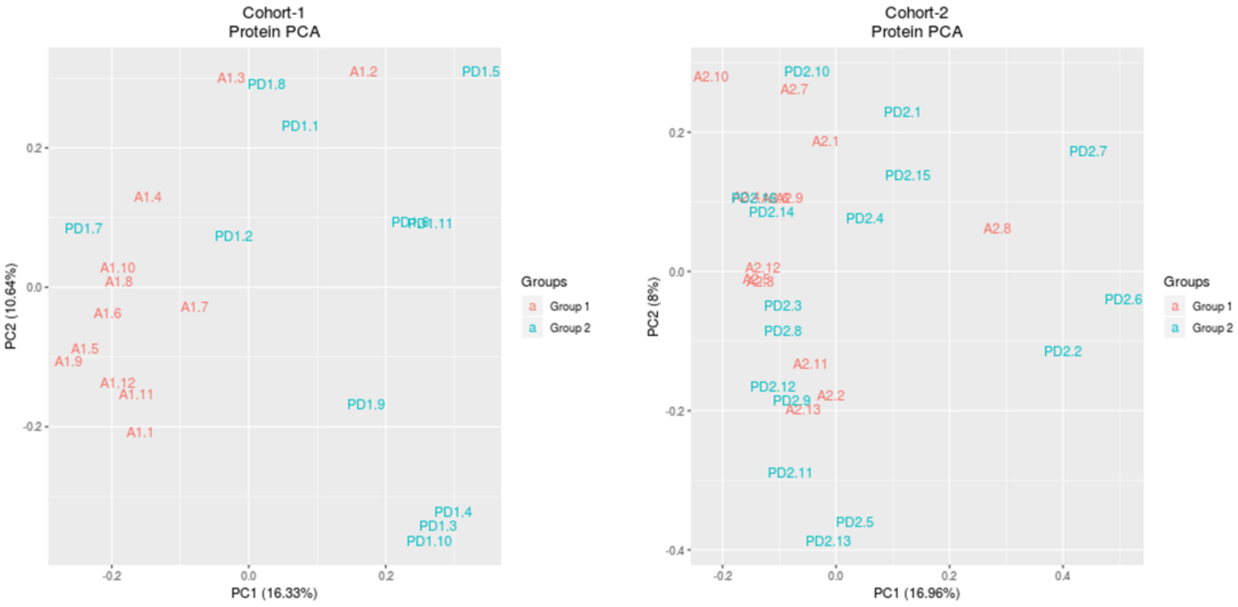

Supplementary Figure S 9. GSEA of differentially expressed human prefrontal cortex grey matter proteins observed in both cohort 1 and cohort 2, Aged (>60 years) versus PD.

(C) Volcano plot showing most enriched molecular functions

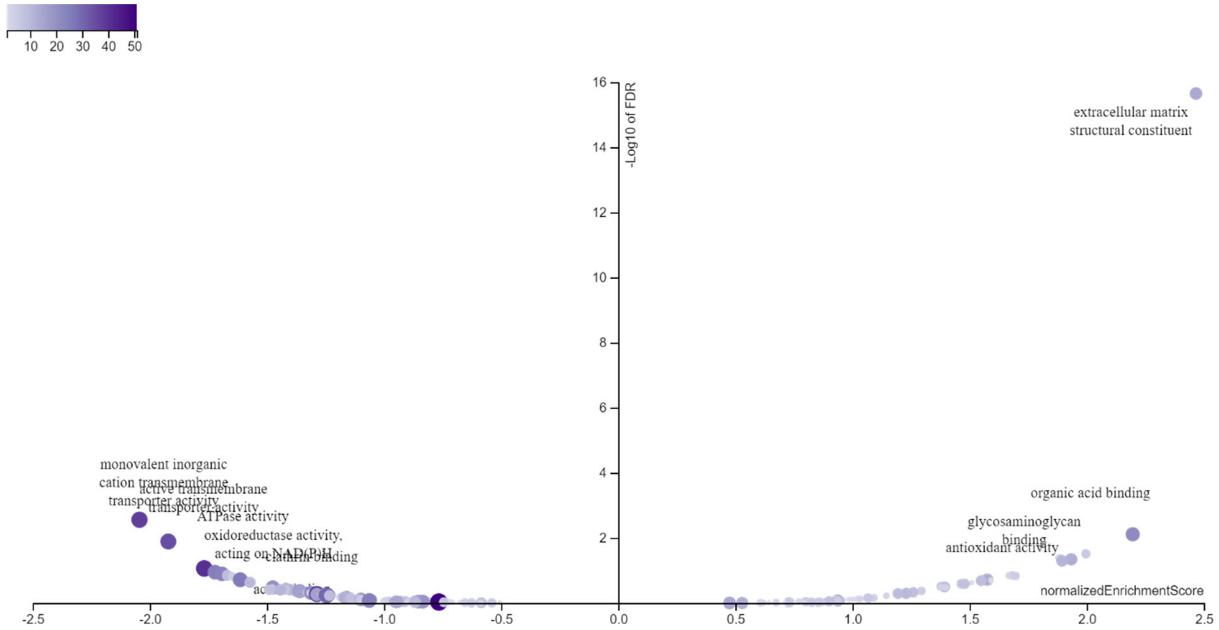

Supplementary Figure S 9. GSEA of differentially expressed human prefrontal cortex grey matter proteins observed in both cohort 1 and cohort 2, Aged (>60 years) versus PD.

(D) Directed acyclic graph of GO terms

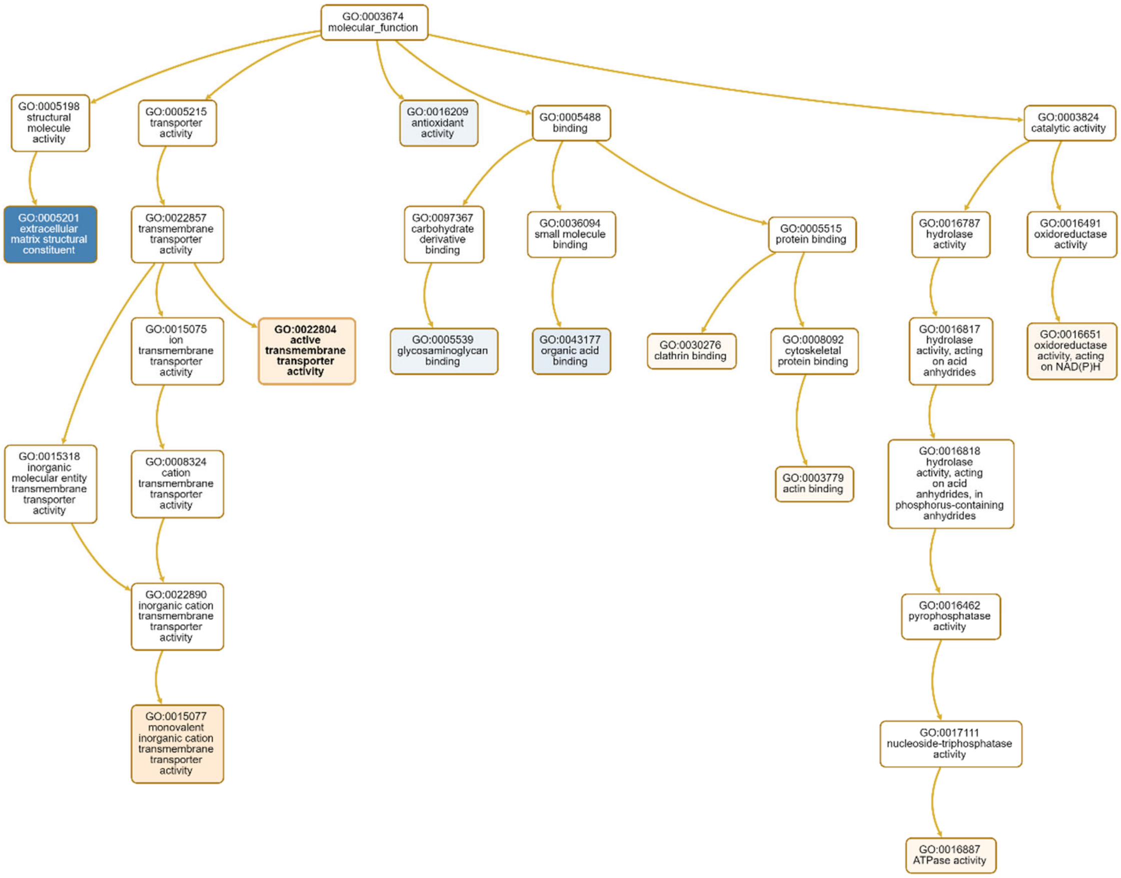

Supplementary Figure S 10. Differential abundance t values for annexins in the human aged vs PD cohorts 1 and 2. Negative values are enriched in PD.

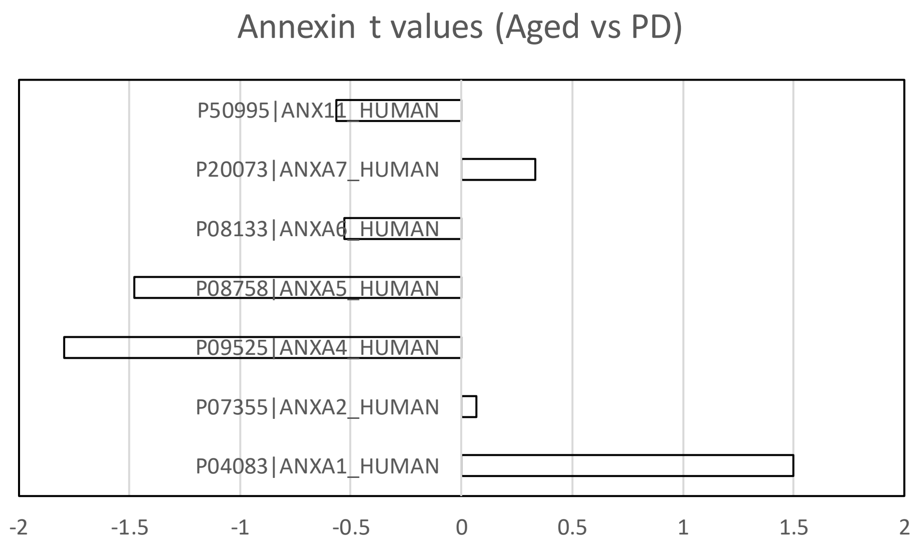

Supplementary Tables

Supplementary Table S 1A. Clinical data for control specimens.

Cohort	Group	BU_ID	Death	SEX	Type of Brain	PMI	Cause of Death
1	Aged	C_0053	69	Male	Control	2	Prostate Cancer
1	Aged	C_0002	73	Male	Control	2	Acute myocardial infarction
1	Aged	C_0050	74	Male	Control	2	Cardiac and/or respiratory failure
1	Aged	C_0060	76	Male	Control	2	Pancreatic Cancer
1	Aged	C_0061	78	Male	Control	3	Cardiac and/or respiratory failure
1	Aged	C_0009	81	Male	Control	3	Cardiac and/or respiratory failure
1	Aged	C_0004	82	Male	Control	2	Cardiorespiratory Arrest
1	Aged	C_0006	86	Male	Control	5	Cardiac Arrest
1	Aged	C_0065	86	Male	Control	2	CHF, Ischemic cardimyopathy
1	Aged	C_0062	87	Male	Control	2	Ruptured Aortic Aneurysm
1	Aged	C_0003	91	Male	Control	2	Metastatic Bladder Cancer
1	Aged	C_0008	91	Male	Control	2	End Stage Renal Disease
2	Young	C_0036	40	Male	Control	17	Cardiac
2	Young	C_0033	43	Male	Control	15	MI
2	Young	C_0037	44	Male	Control	28	Cardiac Arrest
2	Young	C_0035	46	Male	Control	21	Cardiac Arrest
2	Young	C_0075	52	Male	control	23	Heart Attack
2	Young	C_0031	53	Male	Control	24	MI
2	Young	C_0069	54	Male	Control	24	Cardiac Arrest/CHF
2	Young	C_0081	55	Male	Control	26	CPA-MI
2	Young	C_0082	57	Male	Control	18	
2	Young	C_0038	57	Male	Control	20	MI
2	Young	C_0077	36	Male	control	21	electrocution
2	Young	C_0020	60	Male	Control	24	MI
2	Aged	C_0087	64	Male	Control	19	
2	Aged	C_0083	66	Male	Control	32	CPN
2	Aged	C_0017	70	Male	Control	21	MI
2	Aged	C_0039	80	Male	Control	15	
2	Aged	C_0029	93	Male	Control	13	Respiratory Failure; COPD
2	Aged	C_0005	97	Male	Control	2	Metastatic Colon Cancer
2	Aged	C_0010	79	Male	Control	2	Cardiac and/or respiratory failure
2	Aged	C_0013	69	Male	Control	15	COPD
2	Aged	C_0024	69	Male	Control	26	MI-CAD
2	Aged	C_0012	66	Male	Control	19	MI
2	Aged	C_0018	66	Male	Control	17	MI
2	Aged	C_0019	73	Male	Control	19	COPD
2	Aged	C_0021	76	Male	Control	26	Cardiac Arrest

Supplementary Table S 1B. Clinical data for Parkinson's Disease specimens.

Cohort	Group	BU_ID	Death	SEX	Motor Onset	Dementa	ALZ Pathology	PMI	Cause of Death
1	PD	P_0006	83	Male		1	absent	2	Pneumonia, hypertension, vascular dementia
1	PD	P_0012	80	Male	69	0	absent	2	Probable MI
1	PD	P_0013	83	Male	79		absent	2	End_Stage PD
1	PD	P_0014	80	Male	55		absent	2	End-Stage Parkinson's disease; inanition
1	PD	P_0015	84	Male	80	1	absent	2	
1	PD	P_0016	88	Male	85	1	absent	2	End-Stage COPD
1	PD	P_0018	81	Male	73	0	absent	2	Small cell carcinoma of lung
1	PD	P_0019	77	Male	73	0	absent	4	Cardiac and/or respiratory failure, severe MI
1	PD	P_0020	64	Male	59	0	absent	4	Complications of lung cancer
1	PD	P_0021	85	Male		1	absent	3	Dementia; PD; DVD
1	PD	P_0034	64	Male	49	1	absent	4	Failure to thrive secondary to PD, dementia, coronary artery diease
1	PD	P_0063	64	Male	53	0	absent	1	
2	PD	P_0031	65	Male			absent	8	
2	PD	P_0030	66	Male	55	0	absent	11	PD
2	PD	P_0130	68	Male	62	1	absent	18	MI
2	PD	P_0134	68	Male	63		mild	23	Cardiac Arrest
2	PD	P_0136	70	Male			mild	25	
2	PD	P_0135	79	Male	66	0	mild	12	Aspiration Pneumonia
2	PD	P_0023	80	Male	60	1	absent	27	Pneumonia/PD
2	PD	P_0026	85	Male	65	0	absent	16	PD
2	PD	P_0029	89	Male	72	0	absent	31	End Stage Parkinson's Disease
2	PD	P_0022	94	Male			absent	9	Respiratory Arrest/PD
2	PD	P_0132	95	Male	80	1	absent	16	Parkinson's/Dementia
2	PD	P_0027	75	Male			absent	7	Cardiac Arrest
2	PD	P_0028	74	Male			absent	15	End Stage Parkinson's Disease; Bladder Infection
2	PD	P_0033	85	Male	71		absent	19	Cardiovascular Failure
2	PD	P_0126	75	Male	61	1	mild	30	Aspiration Pneumonia
2	PD	P_0133	74	Male	66	0	absent	23	Parkinson's disease

Supplementary Table S 2A. Proteins from the organic acid binding gene set enriched in aged rat striatum from previously published data[4].

ID: GO:0043177; Name: organic acid binding			
Size=27; leadingEdgeNum=11; enrichmentScore=0.61; normalizedEnrichmentScore=2.01; PValue=0.000e+0; FDR=9.763e-3			
Gene Symbol	Gene Name	Entrez Gene	Score
Pgd	phosphogluconate dehydrogenase	100360180	2.9471
Vcan	versican	114122	5.5605
Ddc	dopa decarboxylase	24311	2.3161
Gad1	glutamate decarboxylase 1	24379	2.3627
Pc	pyruvate carboxylase	25104	2.2401
Bcan	brevican	25393	1.9487
Hapln1	hyaluronan and proteoglycan link protein 1	29331	7.21
C1qbp	complement C1q binding protein	29681	5.0672
Arhgdia	Rho GDP dissociation inhibitor alpha	360678	1.9229
Ddah1	dimethylarginine dimethylaminohydrolase 1	64157	2.2448
Rida	reactive intermediate imine deaminase A homolog	65151	3.8715

Supplementary Table S 2B. Proteins from the structural constituent of ribosome gene set enriched in aged rat substantia nigra from previously published data[4].

ID: GO:0003735; Name: structural constituent of ribosome			
Size=23; leadingEdgeNum=13; enrichmentScore=-0.65; normalizedEnrichmentScore=-2.14; PValue=0.000e+0; FDR=1.216e-3			
Gene Symb	Gene Name	Entrez Gene	Score
Rps15a	ribosomal protein S15a	117053	-1.5369
Rps16	ribosomal protein S16	140655	-2.7239
Rpl15	ribosomal protein L15	245981	-1.6261
Rpsa	ribosomal protein SA	29236	-1.913
Rps14	ribosomal protein S14	29284	-3.3179
Rps3a	ribosomal protein S3a	29288	-1.783
Rps8	ribosomal protein S8	65136	-2.1854
Rpl10a	ribosomal protein L10A	81729	-2.2221
Rpl10	ribosomal protein L10	81764	-4.5484
Rpl13	ribosomal protein L13	81765	-2.2695
Rps24	ribosomal protein S24	81776	-2.7733
Rps2	ribosomal protein S2	83789	-2.0951
Rps27	ribosomal protein S27	94266	-1.5909

Supplementary Table S 3. Proteins from the human magnesium ion binding gene set enriched in aged human prefrontal cortex.

ID: GO:0000287; Name: magnesium ion binding			
Size=45; leadingEdgeNum=13; enrichmentScore=0.54; normalizedEnrichmentScore=1.97; PValue=0.000e+0; FDR=4.604e-2			
Gene Symbol	Gene Name	Entrez Gene	Score
PDCD6	programmed cell death 6	10016	2.4941
FARSB	phenylalanyl-tRNA synthetase subunit beta	10056	2.6126
DUT	deoxyuridine triphosphatase	1854	1.9156
ENO1	enolase 1	2023	4.1142
ENO2	enolase 2	2026	5.5922
GNAI1	G protein subunit alpha i1	2770	2.9079
NME1	NME/NM23 nucleoside diphosphate kinase 1	4830	1.4799
PPA1	pyrophosphatase (inorganic) 1	5464	2.691
PRPSAP1	phosphoribosyl pyrophosphate synthetase associated protein 1	5635	2.2597
RAN	RAN, member RAS oncogene family	5901	7.7422
RAP2A	RAP2A, member of RAS oncogene family	5911	2.0748
RHEB	Ras homolog, mTORC1 binding	6009	1.6632
SNCA	synuclein alpha	6622	2.2621

Supplementary Table S 4A. Proteins from the extracellular matrix structural component gene set enriched in human prefrontal cortex comparing unaffected aged versus PD specimens.

ID: GO:0005201; Name: extracellular matrix structural constituent				
Size=21; leadingEdgeNum=14; enrichmentScore=0.69; normalizedEnrichmentScore=2.47; PValue=0.000e+0; FDR=0.000e+0				
User ID	Gene Symbol	Gene Name	Entrez Gene	Score
1277	COL1A1	collagen type I alpha 1 chain	1277	2.7486
1278	COL1A2	collagen type I alpha 2 chain	1278	2.4472
1280	COL2A1	collagen type II alpha 1 chain	1280	2.4159
1281	COL3A1	collagen type III alpha 1 chain	1281	1.7167
1282	COL4A1	collagen type IV alpha 1 chain	1282	1.7107
1284	COL4A2	collagen type IV alpha 2 chain	1284	1.384
1293	COL6A3	collagen type VI alpha 3 chain	1293	1.9479
1404	HAPLN1	hyaluronan and proteoglycan link protein 1	1404	1.0703
1462	VCAN	versican	1462	1.9737
2243	FGA	fibrinogen alpha chain	2243	0.9566
2244	FGB	fibrinogen beta chain	2244	0.948
2266	FGG	fibrinogen gamma chain	2266	1.1769
3371	TNC	tenascin C	3371	2.3006
64129	TINAGL1	tubulointerstitial nephritis antigen like 1	64129	1.2354

Supplementary Table S 4B. Proteins from the organic acid binding gene set enriched in human prefrontal cortex comparing unaffected aged versus PD specimens.

ID: GO:0043177; Name: organic acid binding				
Size=40; leadingEdgeNum=22; enrichmentScore=0.51; normalizedEnrichmentScore=2.20; PValue=0.000e+0; FDR=7.733e-3				
User ID	Gene Symbol	Gene Name	Entrez Gene	Score
10247	RIDA	reactive intermediate imine deaminase A homolog	10247	1.4752
128	ADH5	alcohol dehydrogenase 5 (class III), chi polypeptide	128	1.9285
1404	HAPLN1	hyaluronan and proteoglycan link protein 1	1404	1.0703
1462	VCAN	versican	1462	1.9737
1463	NCAN	neurocan	1463	0.9841
2171	FABP5	fatty acid binding protein 5	2171	0.922
23564	DDAH2	dimethylarginine dimethylaminohydrolase 2	23564	0.888
2571	GAD1	glutamate decarboxylase 1	2571	1.999
3039	HBA1	hemoglobin subunit alpha 1	3039	1.9761
3043	HBB	hemoglobin subunit beta	3043	2.6973
3047	HBG1	hemoglobin subunit gamma 1	3047	1.2441
3048	HBG2	hemoglobin subunit gamma 2	3048	1.2441
4099	MAG	myelin associated glycoprotein	4099	1.1416
445	ASS1	argininosuccinate synthase 1	445	2.4563
5660	PSAP	prosaposin	5660	2.0956
5730	PTGDS	prostaglandin D2 synthase	5730	1.397
5836	PYGL	glycogen phosphorylase L	5836	0.8199
5917	RARS	arginyl-tRNA synthetase	5917	1.7961
60484	HAPLN2	hyaluronan and proteoglycan link protein 2	60484	1.2884
6262	RYR2	ryanodine receptor 2	6262	1.5447
63827	BCAN	brevican	63827	1.1992
9380	GRHPR	glyoxylate and hydroxypyruvate reductase	9380	1.8283

Supplementary Table S 5. Proteins from the glycosaminoglycan binding gene set enriched in prefrontal cortex comparing unaffected aged versus PD specimens.

ID: GO:0005539; Name: glycosaminoglycan binding				
Size=19; leadingEdgeNum=13; enrichmentScore=0.55; normalizedEnrichmentScore=1.93; PValue=0.000e+0; FDR=4.551e-2				
User ID	Gene Symbol	Gene Name	Entrez Gene	Score
1404	HAPLN1	hyaluronan and proteoglycan link protein 1	1404	1.0703
1462	VCAN	versican	1462	1.9737
1463	NCAN	neurocan	1463	0.9841
1809	DPYSL3	dihydropyrimidinase like 3	1809	0.5917
307	ANXA4	annexin A4	307	1.7921
308	ANXA5	annexin A5	308	1.4768
309	ANXA6	annexin A6	309	0.5305
351	APP	amyloid beta precursor protein	351	1.5335
5048	PAFAH1B1	platelet activating factor acetylhydrolase 1b regulatory subunit 1	5048	1.3738
5621	PRNP	prion protein	5621	0.7385
5802	PTPRS	protein tyrosine phosphatase, receptor type S	5802	0.5632
60484	HAPLN2	hyaluronan and proteoglycan link protein 2	60484	1.2884
63827	BCAN	brevican	63827	1.1992

Supplementary Table S 6. Proteins from the monovalent inorganic cation transmembrane transporter activity gene set enriched in prefrontal cortex comparing unaffected aged versus PD specimens.

ID: GO:0015077; Name: monovalent inorganic cation transmembrane transporter activity				
Size=58; leadingEdgeNum=38; enrichmentScore=-0.56; normalizedEnrichmentScore=-2.04; PValue=0.000e+0; FDR=2.768e-3				
User ID	Gene Symbol	Gene Name	Entrez Gene	Score
10476	ATP5PD	ATP synthase peripheral stalk subunit d	10476	-1.8608
1327	COX4I1	cytochrome c oxidase subunit 4I1	1327	-2.541
1337	COX6A1	cytochrome c oxidase subunit 6A1	1337	-2.8616
1345	COX6C	cytochrome c oxidase subunit 6C	1345	-1.7636
23530	NNT	nicotinamide nucleotide transhydrogenase	23530	-1.2948
388662	SLC6A17	solute carrier family 6 member 17	388662	-1.7863
3954	LETM1	leucine zipper and EF-hand containing transmembrane protein 1	3954	-1.4236
4697	NDUFA4	NDUFA4, mitochondrial complex associated	4697	-1.155
478	ATP1A3	ATPase Na+/K+ transporting subunit alpha 3	478	-1.6018
479	ATP12A	ATPase H+/K+ transporting non-gastric alpha2 subunit	479	-1.238
482	ATP1B2	ATPase Na+/K+ transporting subunit beta 2	482	-1.1537
488	ATP2A2	ATPase sarcoplasmic/endoplasmic reticulum Ca2+ transporting 2	488	-2.7324
506	ATP5F1B	ATP synthase F1 subunit beta	506	-1.941
509	ATP5F1C	ATP synthase F1 subunit gamma	509	-2.1328
51382	ATP6V1D	ATPase H+ transporting V1 subunit D	51382	-1.0793
515	ATP5PB	ATP synthase peripheral stalk-membrane subunit b	515	-1.54
51606	ATP6V1H	ATPase H+ transporting V1 subunit H	51606	-1.4659
523	ATP6V1A	ATPase H+ transporting V1 subunit A	523	-3.1106
5250	SLC25A3	solute carrier family 25 member 3	5250	-2.0836
526	ATP6V1B2	ATPase H+ transporting V1 subunit B2	526	-1.1876
528	ATP6V1C1	ATPase H+ transporting V1 subunit C1	528	-1.5198
529	ATP6V1E1	ATPase H+ transporting V1 subunit E1	529	-2.0498
535	ATP6V0A1	ATPase H+ transporting V0 subunit a1	535	-1.5103
537	ATP6AP1	ATPase H+ transporting accessory protein 1	537	-1.8737
539	ATP5PO	ATP synthase peripheral stalk subunit OSCP	539	-1.969
57030	SLC17A7	solute carrier family 17 member 7	57030	-1.641
57282	SLC4A10	solute carrier family 4 member 10	57282	-2.8476
57468	SLC12A5	solute carrier family 12 member 5	57468	-1.0233
6506	SLC1A2	solute carrier family 1 member 2	6506	-2.3548
6507	SLC1A3	solute carrier family 1 member 3	6507	-1.887
6543	SLC8A2	solute carrier family 8 member A2	6543	-1.4728
6616	SNAP25	synaptosome associated protein 25	6616	-3.5623
79751	SLC25A22	solute carrier family 25 member 22	79751	-1.6384
8514	KCNAB2	potassium voltage-gated channel subfamily A regulatory beta subu	8514	-1.3135
8671	SLC4A4	solute carrier family 4 member 4	8671	-2.3251
9114	ATP6V0D1	ATPase H+ transporting V0 subunit d1	9114	-1.7036
9296	ATP6V1F	ATPase H+ transporting V1 subunit F	9296	-1.2432
9377	COX5A	cytochrome c oxidase subunit 5A	9377	-0.9858

Supplementary Table S 7. Proteins from the active transmembrane transporter activity gene set negatively enriched in prefrontal cortex comparing unaffected aged versus PD specimens.

ID: GO:0022804; Name: active transmembrane transporter activity				
Size=54; leadingEdgeNum=35; enrichmentScore=-0.53; normalizedEnrichmentScore=-1.92; PValue=0.000e+0; FDR=1.292e-2				
10476	ATP5PD	ATP synthase peripheral stalk subunit d	10476	-1.8608
292	SLC25A5	solute carrier family 25 member 5	292	-1.4848
388662	SLC6A17	solute carrier family 6 member 17	388662	-1.7863
3954	LETM1	leucine zipper and EF-hand containing transmembrane protein 1	3954	-1.4236
478	ATP1A3	ATPase Na+/K+ transporting subunit alpha 3	478	-1.6018
479	ATP12A	ATPase H+/K+ transporting non-gastric alpha2 subunit	479	-1.238
482	ATP1B2	ATPase Na+/K+ transporting subunit beta 2	482	-1.1537
488	ATP2A2	ATPase sarcoplasmic/endoplasmic reticulum Ca2+ transporting 2	488	-2.7324
490	ATP2B1	ATPase plasma membrane Ca2+ transporting 1	490	-1.4004
491	ATP2B2	ATPase plasma membrane Ca2+ transporting 2	491	-1.4387
492	ATP2B3	ATPase plasma membrane Ca2+ transporting 3	492	-2.0022
493	ATP2B4	ATPase plasma membrane Ca2+ transporting 4	493	-2.1314
506	ATP5F1B	ATP synthase F1 subunit beta	506	-1.941
509	ATP5F1C	ATP synthase F1 subunit gamma	509	-2.1328
51382	ATP6V1D	ATPase H+ transporting V1 subunit D	51382	-1.0793
515	ATP5PB	ATP synthase peripheral stalk-membrane subunit b	515	-1.54
51606	ATP6V1H	ATPase H+ transporting V1 subunit H	51606	-1.4659
523	ATP6V1A	ATPase H+ transporting V1 subunit A	523	-3.1106
5250	SLC25A3	solute carrier family 25 member 3	5250	-2.0836
526	ATP6V1B2	ATPase H+ transporting V1 subunit B2	526	-1.1876
528	ATP6V1C1	ATPase H+ transporting V1 subunit C1	528	-1.5198
529	ATP6V1E1	ATPase H+ transporting V1 subunit E1	529	-2.0498
535	ATP6V0A1	ATPase H+ transporting V0 subunit a1	535	-1.5103
537	ATP6AP1	ATPase H+ transporting accessory protein 1	537	-1.8737
539	ATP5PO	ATP synthase peripheral stalk subunit OSCP	539	-1.969
57030	SLC17A7	solute carrier family 17 member 7	57030	-1.641
57282	SLC4A10	solute carrier family 4 member 10	57282	-2.8476
6506	SLC1A2	solute carrier family 1 member 2	6506	-2.3548
6507	SLC1A3	solute carrier family 1 member 3	6507	-1.887
6509	SLC1A4	solute carrier family 1 member 4	6509	-3.0523
6543	SLC8A2	solute carrier family 8 member A2	6543	-1.4728
79751	SLC25A22	solute carrier family 25 member 22	79751	-1.6384
8671	SLC4A4	solute carrier family 4 member 4	8671	-2.3251
9114	ATP6V0D1	ATPase H+ transporting V0 subunit d1	9114	-1.7036
9296	ATP6V1F	ATPase H+ transporting V1 subunit F	9296	-1.2432

Supplementary References

1 Raghunathan, R., Sethi, M. & Zaia, J. On-slide tissue digestion for mass spectrometry based glycomic and proteomic profiling. *MethodsX* **6**, 2329-2347, doi:https://doi.org/10.1016/j.mex.2019.09.029 (2019).

2 Esko, J. D. & Selleck, S. B. Order out of chaos: assembly of ligand binding sites in heparan sulfate. *Annu Rev Biochem* **71**, 435-471 (2002).

3 Lindahl, U. & Li, J. P. Interactions between heparan sulfate and proteins-design and functional implications. *Int Rev Cell Mol Biol* **276**, 105-159, doi:10.1016/S1937-6448(09)76003-4 (2009).

4 Raghunathan, R. *et al.* Glycomic and Proteomic Changes in Aging Brain Nigrostriatal Pathway. *Molecular & Cellular Proteomics* **17**, 1778-1787, doi:10.1074/mcp.RA118.000680 (2018).
